# The Multifaceted Effects of Agmatine on Functional Recovery after Spinal Cord Injury through Modulations of BMP-2/4/7 Expressions in Neurons and Glial Cells

**DOI:** 10.1371/journal.pone.0053911

**Published:** 2013-01-21

**Authors:** Yu Mi Park, Won Taek Lee, Kiran Kumar Bokara, Su Kyoung Seo, Seung Hwa Park, Jae Hwan Kim, Midori A. Yenari, Kyung Ah Park, Jong Eun Lee

**Affiliations:** 1 Department of Anatomy, Yonsei University College of Medicine, Seoul, Republic of Korea; 2 BK 21 Project for Medical Science, Yonsei University College of Medicine, Seoul, Republic of Korea; 3 Department of Anatomy, Konkuk University College of Medicine, Seoul, Republic of Korea; 4 Department of Neurology, University of California San Francisco and Veterans Affairs Medical Center, San Francisco, California, United States of America; Max Planck Institute of Psychiatry, Germany

## Abstract

Presently, few treatments for spinal cord injury (SCI) are available and none have facilitated neural regeneration and/or significant functional improvement. Agmatine (Agm), a guanidinium compound formed from decarboxylation of L-arginine by arginine decarboxylase, is a neurotransmitter/neuromodulator and been reported to exert neuroprotective effects in central nervous system injury models including SCI. The purpose of this study was to demonstrate the multifaceted effects of Agm on functional recovery and remyelinating events following SCI. Compression SCI in mice was produced by placing a 15 g/mm^2^ weight for 1 min at thoracic vertebra (Th) 9 segment. Mice that received an intraperitoneal (i.p.) injection of Agm (100 mg/kg/day) within 1 hour after SCI until 35 days showed improvement in locomotor recovery and bladder function. Emphasis was made on the analysis of remyelination events, neuronal cell preservation and ablation of glial scar area following SCI. Agm treatment significantly inhibited the demyelination events, neuronal loss and glial scar around the lesion site. In light of recent findings that expressions of bone morphogenetic proteins (BMPs) are modulated in the neuronal and glial cell population after SCI, we hypothesized whether Agm could modulate BMP- 2/4/7 expressions in neurons, astrocytes, oligodendrocytes and play key role in promoting the neuronal and glial cell survival in the injured spinal cord. The results from computer assisted stereological toolbox analysis (CAST) demonstrate that Agm treatment dramatically increased BMP- 2/7 expressions in neurons and oligodendrocytes. On the other hand, BMP- 4 expressions were significantly decreased in astrocytes and oligodendrocytes around the lesion site. Together, our results reveal that Agm treatment improved neurological and histological outcomes, induced oligodendrogenesis, protected neurons, and decreased glial scar formation through modulating the BMP- 2/4/7 expressions following SCI.

## Introduction

Spinal cord injury (SCI) often results in permanent disability or loss of movement (paralysis) and sensation below the site of injury leading either to paraplegia (thoracic level injury) or tetraplegia (cervical level injury) [1]. SCI rostral to the lumbosacral level disrupts voluntary and supraspinal control of voiding and induces a considerable reorganization of the micturition reflex pathway. The urinary bladder is initially are flexic, but then becomes hyperreflexic because of the emergence of spinal micturition reflex pathway following SCI [2]. SCI leads to neuronal and glial cell death, induces glial scar formation [3] and inhibits axonal regeneration and remyelination [4]. Oligodendrocytes produce myelin that wraps around the axons of neurons to enable them to conduct electrical impulses [5], [6] and neurotrophic factors to support the maintenance of nerve cells. Oligodendrocytes are lost during SCI, resulting in the loss of myelin and motor function that cause paralysis in animals.

Agmatine (Agm) (4-aminobutyl guanidine), NH_2_-CH_2_-CH_2_-CH_2_-CH_2_-NH-C (-NH_2_) ( = NH), is an endogenous amine and four carbon guanidine compound formed by decarboxylation of arginine [7]. Agm was implicated in modulation of neurotransmission functions. It interacts with various neurotransmitter receptors, including nicotine, N-methyl-d-aspartate (NMDA) α_2_-adrenoceptors and imidazoline receptors [7], [8]. In addition, this molecule can interfere with second messenger pathways by acting as an adenosine diphosphate (ADP)-ribose acceptor thereby inhibiting ADP-ribosylation of proteins [9]. Exogenous administration of Agm significantly reduces pain induced by inflammation following SCI [10]. The above characteristics of Agm led us to hypothesize that it might serve as a neuroprotective agent following neurotrauma.

Bone morphogenetic proteins (BMPs) are multifunctional growth factors that belong to the transforming growth factor-β (TGF-β) super family. BMPs signal through serine/threonine kinase receptors, composed of type I and II BMP receptors [11]. BMPs play important roles as trophic factors that may act in cell death regulation/differentiation [12], proliferation of neural progenitor cells and are also involved in restoration of injured neurons following various central nervous system (CNS) injuries [12], [13]. Among the various types of BMPs, BMP- 2/7 in particular promotes differentiation and boosts dendrite growth in cultured striatal neurons [14] and modulates the balance between glial cells and neurons [15]. Earlier reports suggested that the BMP levels are altered following SCI [16]. BMP- 7 given intravenously showed neuroprotective effects following SCI [17], [18]. Furthermore, BMP- 4 signaling was reported to be essential for astrocytes lineage proliferation following SCI [19]. Conversely, disruption of BMP signaling in vivo negatively affects astrogliogenesis [20]. Several groups have studied the effects of BMP signaling after SCI with mixed results. It was also demonstrated that BMP signaling enhances axonal outgrowth and locomotor recovery after SCI. These observations suggest that BMP signaling may be involved in both the beneficial and the detrimental effects following SCI [21], [22]. Agm treatment following SCI was shown to improve locomotor functions and reduce collagen scar formation accompanied with TGF-β and BMP- 7 expressions suggesting that BMPs may regulate neural cell lineage commitment in vivo [23]. Based on the previous evidences reporting the beneficial effects of Agm, we hypothesized that Agm treatment, a well-known neuroprotector, may have effects on (1) recovery of locomotory and physiological functions, (2) facilitate axonal remyelination, (3) promote protection of neurons, (4) attenuate glial scar formation, and (5) modulate the BMP- 2/4/7 expressions in neuronal and glial cells following SCI. In this study, the mice subjected to SCI were divided into agmatine treatment group (Agm treated group) and saline treatment group (EC group) along with parallel controls. All the experimental groups were examined for functional recovery and urinary bladder functions which included open field test and urine residual volume measurement respectively following SCI. Histological sections were examined to measure glial scar using an imaging program and the axonal remyelination was confirmed with myelin basic proteins (MBPs) staining. The surviving neurons, oligodendrocytes, and astrocytes were confirmed by counting the total cell numbers of microtubule-associated protein-2 (MAP-2), oligodendrocyte transcription factor-2 (Olig-2), and glial fibrillary acidic protein (GFAP) immunopositive cells in the total spinal cord (Th 8–Th 10 segments) and also in the rostral (Th 8), lesion (Th 9) and caudal regions (Th 10) separately using computer assisted sterological toolbox analysis (CAST) following SCI.

This first study provides robust evidence of the beneficial effects of Agm treatment leading to lasting improvements of structure and function through modulating the BMP- 2/4/7 expressions in neurons, oligodendrocytes, and astrocytes, which could be vital for directing the axonal remyelination and protect damaged neurons following SCI.

## Materials and Methods

### Animals

Studies were conducted on male Imprinting Control Region (ICR) mice, 8 weeks old, weighing 28±5 g (Sam tako, South Korea). All animal experiments were performed in accordance with the Korean Food and Drug Administration (KFDA) guidelines. Protocols were reviewed and approved by the Institutional Animal Care and Use Committee (IACUC) of the Yonsei Laboratory Animal Research Center (YLARC) (Permit #: 10-114). All mice were maintained in the specific pathogen-free facility of the YLARC.

### Compression Spinal Cord Injury (SCI) and Agmatine (Agm) Treatment

The mice (*n* = 280) were anesthetized with a combination of ketamine (100 mg/kg) and xylazine (rompun) (10 mg/kg) by intraperitoneal (i.p.) injection. Body temperatures of the animals were monitored with rectal probes maintained between 36.5°C and 37.5°C with heating pads and lamps. A laminectomy of thoracic vertebra (Th) segment 8– Th 10 (potentially Th 9 as a result of surgical variability) was performed using a fine pair of rongers without damaging the durameter. The spinal cord at Th 9 was injured with a bilateral micro clamp clip (Fine Science Tools Inc in Foster City, CA, USA). A compression of 15 g/mm^2^ was applied to the exposed spinal cord for 1 minute. The clip was then removed and the skin sutured. Bladders were manually pressed twice daily until spontaneous voiding occurred, and any hematuria or urinary tract infection was treated with ampicillin (1 mg/kg) daily for one week. Food and water were freely accessible at a lowered height in their cages. Agmatine (Agm treated group, *n* = 101) was administered at a dose 100 mg/kg/day (Sigma) by intraperitoneal injection within 1 hour after SCI until 35 days post injury (DPI). Experimental control mice (EC group, *n* = 101) received intraperitoneal (i.p.) injections of saline instead of Agm. Normal control mice without spinal cord injury (NC group, *n* = 78) were maintained all through the experiment (see Table S1).

### Behavior Test

Mice were tested for locomotor deficits at 1 day, 3 days, 7 days, 10 days, 14 days (*n* = 30, per group), 21 days, 28 days and 35 days (*n* = 15, per group) after SCI in an open field using the Basso Mouse Scale **(**BMS) as previously described [24], [25] (see Table S1). The BMS test scores were rated on a scale of 0 to 9 with 0 being no function (complete paralysis of the hind limbs) and 9 being normal (normal movement of the hind limbs). The performance of both left and right limbs of the mice were assessed depending on the average BMS test scale scores obtained until 35 DPI.

### Bladder Function Analysis

Assessment of bladder function was carried in all the experimental groups until 14 DPI. The bladders were manually stimulated twice a day until 14 days (*n* = 10, per group) till they regained to normal autonomic bladder function (approximately 11–15 DPI) (see Table S1). Retained urine from each mouse was collected and measured from all the experimental groups both in the morning and evening sessions (12 hours interval) until 14 DPI [26].

### Ultra Structural Studies of Spinal Cord using Transmission Electron Microscope

Transmission electron microscopic studies were done to assess micro structural changes of myelin sheath after SCI. Briefly, mice were perfused with normal saline followed by a solution containing 2% glutaraldehyde - 4% paraformaldehyde. After thermal stresses for 12 hours, each sample was fixed with 2% glutaraldehyde - paraformaldehyde in 0.1 M phosphate buffer (PB), pH 7.4 for 2 hour and washed three times for 30 min in 0.1 M PB. They were post fixed with 1% OsO_4_ dissolved in 0.1 M PB for 2 hour and dehydrated in ascending gradual series (50–100%) of ethanol and infiltrated with propylene oxide. Specimens were embedded by Poly/Bed 812 kit (Polysciences). After embedding in pure fresh resin they were polymerized at 60°C in electron microscope oven (TD-700, DOSAKA, Japan) for 24 hours. After incubation, 350 nm thick sections were initially cut and stained with toluidine blue for light microscope to confirm the quality of embedding. Later, 70 nm thin sections were cut by LEICA Ultracut UCT Ultra-microtome (Leica Microsystems, Austria) and were double stained with 7% uranyl acetate for 20 min and lead citrate for contrast staining. All of the thin sections were observed under transmission electron microscopy (JEM-1011, JEOL, Japan) at the acceleration voltage of 80 kv.

### Luxol Fast Blue Staining

To confirm the myelination at the injury site before and after Agm treatment, the luxol fast blue staining was performed. The spinal cord tissue section (20 µm) from NC, EC and Agm treated groups were placed directly into 1∶1 alcohol/chloroform for a few hours/overnight and then were hydrated back with 95% ethyl alcohol. Later the tissue slides were incubated with luxol fast blue stain for 16 hrs at 56°C in oven and then excess stain was rinsed with 95% alcohol. The tissue sections were counterstained with cresyl violet solution for 30–40 seconds and mounted onto slides.

### Tissue Processing for Immunohistochemistry

At 1, 7, 14 and 35 DPI, mice were deeply anaesthetized with a combination of ketamine (100 mg/kg) and xylazine (rompun) (10 mg/kg) and were perfused transcardially with 0.9% saline containing 0.1% heparin, followed by 4% paraformaldehyde (PFA). The spinal cords were removed and incubated in the same fixative for 2 h at room temperature and then cryoprotected in 30% sucrose PBS solution overnight. A segment of each cord, extending from 3 mm rostral to 3 mm caudal to the lesion site representing Th 8 - Th 10 segments of the spinal cord was embedded in medium (Tissue - Tek O.C.T. compound, Sakura Finetek USA, Inc., Torrance, CA, USA). Serial transverse sections were cut at 20 µm for immmunohistochemistry (*n* = 18, per group) and 40 µm (*n* = 60, per group) for CAST analysis on a cryostat and mounted onto slides (ColorFrost/Plus; Fisher, Pittsburgh, PA, USA) (see Table S1).

### GFAP Positive Area Measurement

To quantify the glial scar area in the EC group (*n* = 4) and the Agm treated group (*n* = 4) (see Table S1), 20 µm thick spinal cord sections were sequentially immunoreacted with GFAP antibody (1∶500, mouse, monoclonal; Thermo) at 4°C overnight. Later on the tissue slides were incubated with appropriate biotinylated secondary antibody. Immunostaining of GFAP was visualized using the ABC kit (Vector, Burlingame, CA, USA), then reacted with 3, 3′– diaminobenzidine tetra hydrochloride (DAB, sigma, St.Lousi, MO, USA). Negative controls were prepared without adding primary antibody. The entire GFAP positive area around the lesion site of the spinal cord both in EC and Agm treated group was measured by computer-associated scanning image analysis system (Optimas ver 6.1, Optimas, Bothwell, WA, USA) for considering the glial scar area.

### 3, 3′- diaminobenzidine Tetra Hydrochloride (DAB) Immunostaining for CAST Analysis

The 40 µm thick spinal cord sections (*n* = 60, per group) (see Table S1) were treated with 10% blocking serum at 37°C for 1 hour. Primary antibodies: anti-mouse GFAP (1∶500, monoclonal antibody; Thermo), anti-mouse MAP-2 (1∶500, monoclonal antibody; Sigma), anti-goat Olig-2 (1∶500, polyclonal antibody; Santa-cruz), anti-mouse BMP-2 (1∶500, monoclonal antibody; Abcam), anti-mouse BMP-4 (1∶250, polyclonal antibody; Santa-cruz), and anti-rabbit BMP-7 (1∶500, monoclonal antibody; Abcam) diluted in antibody diluents solution (Invitrogen, Carlsbd, CA) were added to the tissue section samples and incubated at 4°C overnight. Primary antibody was removed and tissue slides were washed 3 times for 3 min each with PBS. Later samples were incubated with appropriate secondary antibody prepared in dilution buffer conjugated to peroxidase (1∶500; Abcam) for 2 h at room temperature. Tissue sections were washed again 3 times for 3 min each with PBS and stained with 3, 3′ - diamino-benzidine tetra hydrochloride (DAB, sigma, St. Louis, MO, USA) and SG substrate kit (Vector, Burlingame, CA, USA) for 5 min at room temperature. Staining with DAB results in the deposition of a brown insoluble precipitate and staining with SG substrate results in blue-gray precipitate at the antigenic sites containing the specific epitopes recognized by the primary antibody and were visualized using light microscope. Immunostaining negative controls were prepared without the primary antibody.

### Computer Assisted Stereological Toolbox (CAST) Analysis

The spinal cord segments (Th 8–Th 10) were obtained from control mice without SCI (NC group), SCI mice treated with saline (EC group) and SCI mice treated with agmatine (Agm treated group). Briefly, 40–50 longitudinal cryotissue sections (40 µm thick) of the spinal cord were obtained from each animal and were collected sequentially on histology slides. Every 5^th^ slide was chosen from the total of 40–50 tissue sections for the CAST analysis. The tissue slides were incubated with respective primary antibodies and washed in PBS three times followed by appropriate biotinylated secondary antibody incubation. The DAB and SG stained antigens in the spinal cord (Th 8–Th 10 segments) were quantified using computer assisted stereological toolbox (CAST stereological analysis of cells) (software Ver. 2.3.1.5, applied to an Olympus BX-51, Melville, NY, USA). CAST is a data collection program for obtaining efficient and unbiased estimates of cell number, cell density, cell size, and other stereological quantities. CAST evaluation was performed using a morphometric system consisting of an Olympus BX-51 microscope equipped with a motorized stage, which was controlled by a computer for manual interactive counting on the computer screen. The software CAST-grid version 2.3.1.5 (Olympus, Albertslund, Denmark) was used to generate counting areas. A counting frame was placed randomly and it was systematically moved throughout the encircled counting area until 20% of the entire area was sampled using a 1,000×objective. The cells showing the plasma membrane, a visible nucleus, located within the counting frame were considered positive. The mean number of immunopositive cells per square millimeter of tissue was assessed from each mouse spinal cord tissue [27].



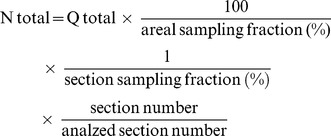



The formula to count the total number of cells using the CAST analysis software program as described by the manufacturer was as follows; N total, represents the total number of immunopositive cells stained per one subject; Q total, a raw count of immunopositive cells per one set per animal group; areal sampling fraction (%), the percentage of the selected area in the total spinal cord (Th 8–Th 10 segments); section sampling fraction (%), the total immunostained area of the selected tissue; section number, the total number of tissue sections prepared from the total spinal cord (Th 8–Th 10 segments); analyzed section number, the number of tissue sections selected for the CAST analysis.

The spinal cord (Th 8–Th 10 segments) was divided into three regions and each segment was identified as: 1) Th 8 is considered as rostral site of the spinal cord from the lesion site, 2) Th 9 is considered as lesion site of the spinal cord and 3) Th 10 is considered as caudal site of the spinal cord from the lesion site.

***Note:*** All experimenters involved with CAST analysis were blinded to treatment group identity.

### Immunofluorescence Studies Using Confocal Microscopy

The frozen sections (*n* = 18, per group) (see Table S1) were air dried at room temperature for 20 min and were blocked for 1 hour using blocking solution at 37°C for 1 hour. The following primary antibodies were used overnight at 4°C: anti-mouse GFAP (1∶500, monoclonal antibody; Thermo), anti-mouse BMP-2 (1∶500, monoclonal antibody; Abcam), anti-mouse BMP-4 (1∶250, polyclonal antibody; Santa-cruz), anti-rabbit BMP-7 (1∶500, monoclonal antibody; Abcam), anti-mouse MAP-2 (1∶500, monoclonal antibody; Sigma), anti-goat Olig-2 (1∶250, polyclonal antibody; Santa-cruz), anti-rat MBP (1∶500, Abcam), anti-mouse Neuronal Nuclei (NeuN) (1∶500, monoclonal antibody; Millipore), and anti-rabbit neurofilament (NF) (1∶500, monoclonal antibody; Millipore), anti-rabbit 5-HT (1∶10000, monoclonal antibody; Abcam), anti-rabbit p53 (1∶500, monoclonal antibody; santa-cruz) and anti-rabbit NG2 (1∶500, monoclonal antibody; Millipore). Then the slides were washed in PBS three times and were incubated with appropriate secondary antibodies conjugated either with fluorescein isothiocyanate **(** *FITC)* (1∶500, Chemicon) or rhodamine (1∶500, Chemicon) at 37°C for 2 hours. The slides were stained with 4′-6-diamidino-2-phenylindole (DAPI) (Vector, Burlingame, USA) to counterstain the nuclei and were coverslipped with mounting medium. Images were taken using a confocal microscopy (LSM700; Zeiss, Thornwood, NY, USA).

### Western Blot Analysis

For immunoblotting, spinal cords from each experimental group (*n* = 20–25, per group) (see Table S1) were dissected, placed in ice-cold artificial CSF (124 mM NaCl, 3 mM KCl, 1 mM NaHPO4, 26 mM NaHCO3, 1.5 mM MgSO4, 1.5 mM CaCl2, and 10 mM glucose), and cleaned from meninges and nerve roots. Spinal cord tissue samples were homogenized on ice using a homogenizer in 500 µL radio immunoprecipitation assay buffer (RIPA) containing 150 mM sodium chloride, 1% nonidet-p40 (NP-40), 0.5% sodium deoxycholate, and 0.1% sodium dodecyl sulphate (SDS), 50 mM Tris (pH 8.0) and phosphatase inhibitor solution, 20 mM/L Tris -HCl (pH 7.5), 2 µg/ml aprotinin, 5 µg/ml leupeptin, 1 µg/ml pepstatin A, 1 mM phenylmethylsulphonyl fluoride (PMSF), 5 mM ethylenediaminetetraacetic acid (EDTA), 1 mM EGTA, 5 mM sodium fluoride (NaF), and 1 mM Sodium Orthovanadate (Na_3_VO_4_). The homogenates were centrifuged at 15,000 g for 15 minutes and protein concentrations were determined by BCA protein assay. Equal amounts of protein (50 µg) were loaded onto 8–10% polyacrylamide gel and were transferred onto PVDF membranes by electrophoresis. The membranes were then blocked with Tris buffered saline with tween -20 (TBST) buffer containing 20 mM Tris-HCI, 5% skim milk, 150 mM NaCI, and 0.05% Tween 20 at pH 7.6, for 1hour at 37°C. The following primary antibodies were used overnight at 4°C: anti-mouse GFAP (1∶1000, monoclonal antibody; Thermo), anti-rabbit S100β (1∶1000, monoclonal antibody; Abcam), anti-mouse BMP-2 (1∶1000, monoclonal antibody; Abcam), anti-mouse BMP-4 (1∶1000, polyclonal antibody; Santa-cruz), anti-rabbit BMP-7 (1∶1000, monoclonal antibody; Abcam), anti-mouse MAP-2 (1∶1000, monoclonal antibody; Sigma), and anti-goat Olig-2 (1∶1000, polyclonal antibody; Santa-cruz). The membranes were washed with TBST and incubated with horseradish peroxidase-conjugated with anti-mouse, anti-rabbit and anti-goat, secondary antibody (1∶2000; Chemicon) at 37°C for 2 hours. Following the incubation, membranes were incubated in super-signal west pico chemiluminescent substrate according to the manufacturer’s specifications. Immunoreactive bands were digitally scanned and analyzed with Image J software (National Institutes of Health). To control for equal protein loading, membranes were also probed for β-actin and the protein values were normalized to β-actin (1∶3000, monoclonal antibody; Abcam).

### Statistical Analysis

All statistical analyses were performed using SPSS 18.0. The data were presented as means and standard errors (S.E.M.).One-way or two-way repeated measures ANOVA was used to compare the experimental groups, with post-hoc Tukey's test for multiple comparisons. Differences were considered statistically significant at *P*<0.05.

## Results

### Agmatine Treatment Enhanced Functional Outcome and Prevented Cell Death Following SCI

**Figure 1:**
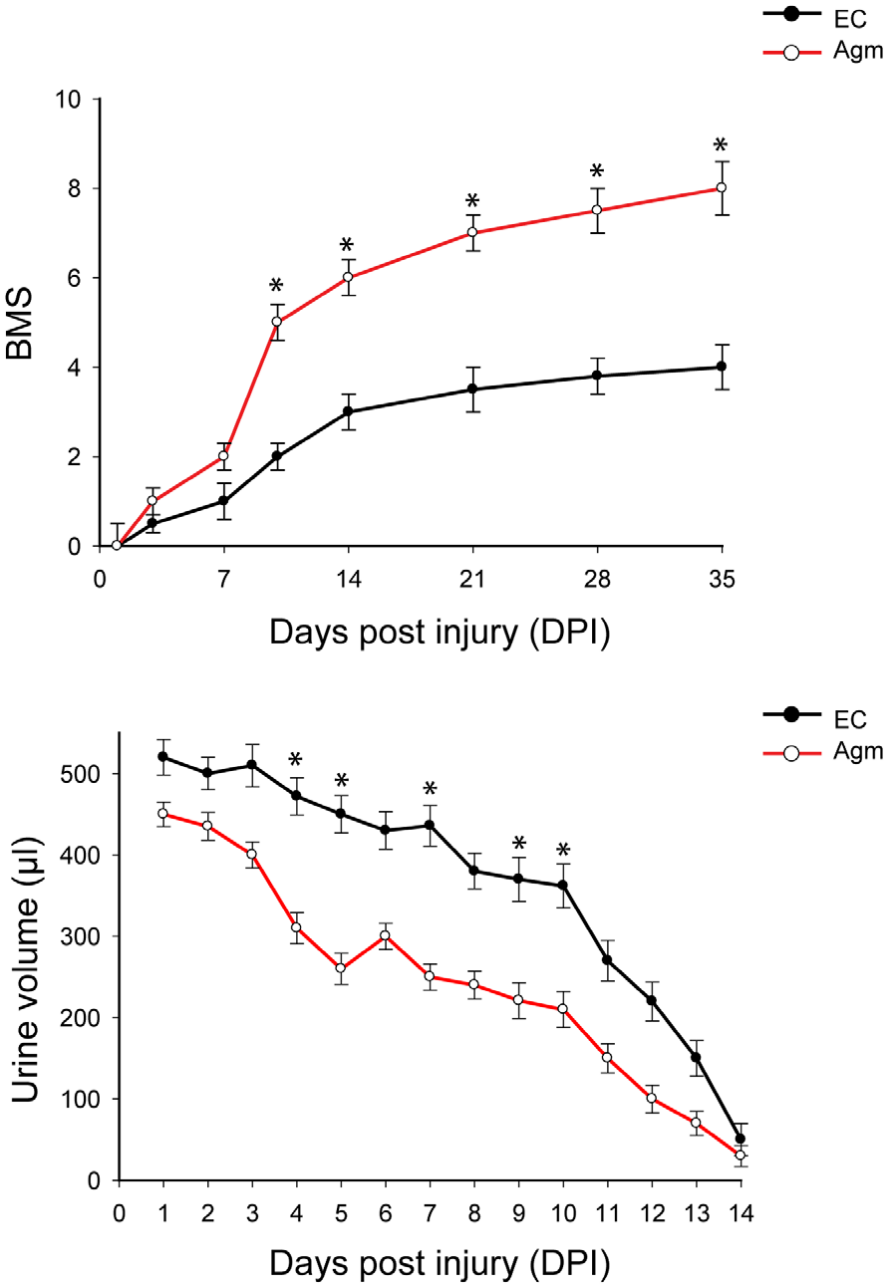
Agmatine treatment enhanced functional outcome following compression SCI. The BMS score was used to evaluate hindlimb locomotor function. The BMS scores in the Agm treated mice (Agm treated group, *n* = 30) were significantly higher than the saline treated mice (EC group, *n* = 30) at 1, 3, 7, 10, 14, 21, 28 and 35 DPI. The Bladder residual urine volumes were measured in Agm treated group (*n* = 10) and in the EC group (*n* = 10). The graph showed that the amount of residual urine was significantly decreased in the Agm treated group compared with the EC group until 14 DPI. *, *p*<0.05 EC group vs Agm treated group. Results represent mean ± S.E.M.

The functional recovery of the mice subjected to SCI was assessed using the Basso Mouse Scale (BMS) scores. After SCI, the EC group (*n* = 30) and Agm treated group (*n* = 30) showed no ankle movement at 1 DPI (BMS score 0). Subsequently, the Agm treated group showed extensive ankle movements (BMS score 2) at 7 DPI and reached frequent or consistent plantar stepping, paws parallel at initial contact and lift off, and severe trunk instability (BMS score 6.8) at 21 DPI. At 35 DPI, the Agm treated group demonstrated frequent or consistent plantar stepping, some or mostly coordinated, paws parallel at initial contact and lift off, and normal trunk stability with up and down tail movement (BMS score 8). Even though the EC group showed occasional plantar stepping but the mice failed to frequent or consistent plantar stepping and their BMS scores were reached only up to a maximum of 4 points at 35 DPI. Our behavioral test results suggested that Agm treated mice showed improvement in the functional recovery compared with that of the saline treated mice (Fig. 1). SCI results in the blockage of the reflex signals between the brain and the bladder and consequently, the self urination fails [2], [28], [29]. Regaining bladder, bowel, and sexual function are some of the highest priorities following SCI and urinary function should be monitored as an outcome measure in SCI models [30], [31]. In this study we assessed whether Agm treatment could improve the bladder function following SCI (*n* = 10, per group). Residual urine was collected and the volumes were measured until 14 DPI. Assessment of bladder function revealed that Agm treatment significantly reduced peak residual urine volumes from 4 day until 10 days compared to saline treated group. This effect of low residual volumes of urine was observed both in the EC and Agm treatment group after 10 DPI and the EC group showed improvement in self voiding and at the end of 14 DPI, both the Agm treated and EC group totally regained the normal voiding function with no urine residual volumes while manual pressing of the bladders. These results depict that Agm treatment could retain the balance between the brain and spinal cord and helps in maintaining the reflex signals for self-urination. This effect was not due to altered fluid intake because fluid consumption did not differ between Agm and saline treated groups (Data not shown). SCI induces a series of endogenous biochemical changes that lead to secondary degeneration, including apoptosis. The p53-mediated apoptosis is likely to be an important mechanism of cell death in SCI [32], [33]. Our immunohistochemical staining results revealed that SCI induced the activation of p53 expression following SCI in the EC group. But fewer number of p53^+^ cells were seen in the lesion site in the Agm treated group compared with EC group both at 14 and 35 days DPI (Fig. S1).

### Agmatine Treatment Promoted Remyelination Following SCI

**Figure 2:**
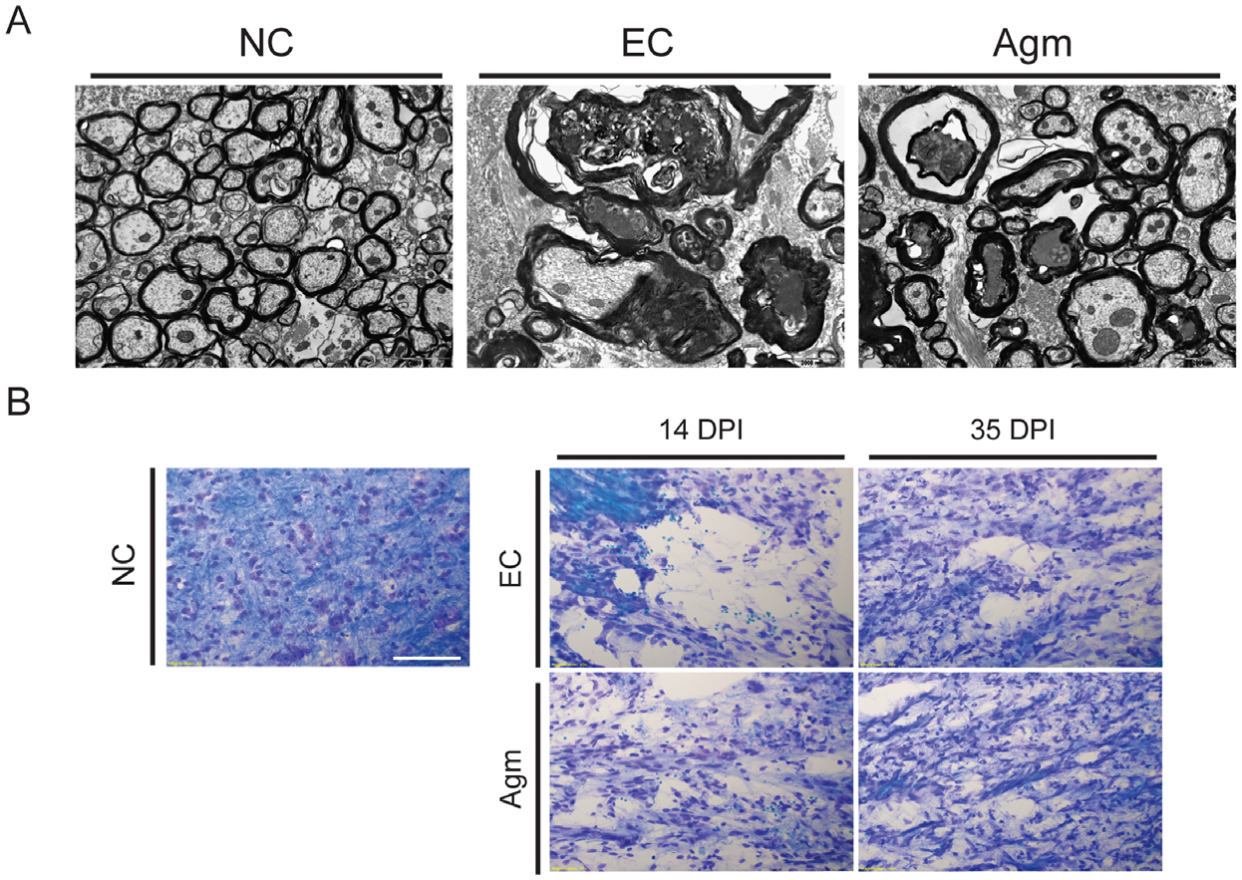
Agmatine treatment promoted remyelination following SCI. (A) Myelin sheath formation in white matter of normal and injured spinal cord was observed by transmission electron microscope (TEM) (1,0000x). The myelination of white matter was ultrastructurally improved with less degenerated myelin in the Agm treated group while swollen axons with broken myelin sheaths were found across the degenerative white matter in the lesioned area in the EC group at 14 DPI. (B) Luxol fast blue staining results showed a remarkable increase of myelin (blue) and neuron (violet) stained cells indicating the reconstruction of lost myelin and neurons in the Agm treated group compared with the EC group at 14 and 35 DPI. Scale bars: 20 µm.

SCI induces local inflammation and demyelination of the white matter around the lesion site, resulting in disrupted axonal conduction. Enhancement of oligodendrocytes or progenitors proliferation to enhance remyelination and functional recovery may lead to repair and restoration of locomotory function after SCI [34]. To ascertain whether Agm could enhance the remyelination process following SCI, transmission electron microscopic studies (TEM) were performed to assess the micro-structural changes of the myelin sheath after SCI. TEM results suggested that Agm treated group (*n* = 3) showed better pattern of myelination in the white matter of lesioned spinal cords compared to EC group (*n* = 3) at 14 days following SCI. In the EC group, a number of large swollen axons with broken myelin sheaths were found across the degenerative white matter in the lesioned area. Thick myelin sheaths broke down and compact layers split apart irregularly and demyelinated axons were amongst the degenerative axons with enlarged spaces between the axolemma and deteriorating sheaths were also prominent in the EC group. Contrast to EC group, Agm treated group showed less broken myelin sheaths and more compact myelination around the lesion site. Moreover, demyelinated axons were found less across the degenerating area in the Agm treated group (Fig. 2A). The myelination was also checked by luxol fast blue staining in NC group, EC group and Agm treated group (*n* = 3, per group) at 14 and 35 DPI. The staining results showed a remarkable increase of myelin (blue) and neurons (violet) stained cells indicating the reconstruction of lost myelin and neurons in the Agm treated group compared with the EC group (Fig. 2B).

Following SCI, the fibers (serotonergic) originated from brain stem which extend the projections to the spinal cord get completely damaged at the lesion site which results in sensorimotor dysfunctions. To determine the effect of Agm (*n* = 3) on regeneration of serotonergic fibers, which are important for the motor functional recovery of hind limbs [35], [36], 5-HT immunostaining was performed in the distal segment of the spinal cord at 14 and 35 DPI. The staining results showed bead like structures with broken morphology of the serotogenic nerve fibers both at 14 and 35 DPI in the EC group (*n* = 3). Conversely Agm treated group showed dense network of serotogenic fibers both at 14 and 35 DPI (Fig. S2) representing the restoration of sensorimotor dysfunctions.

**Figure 3:**
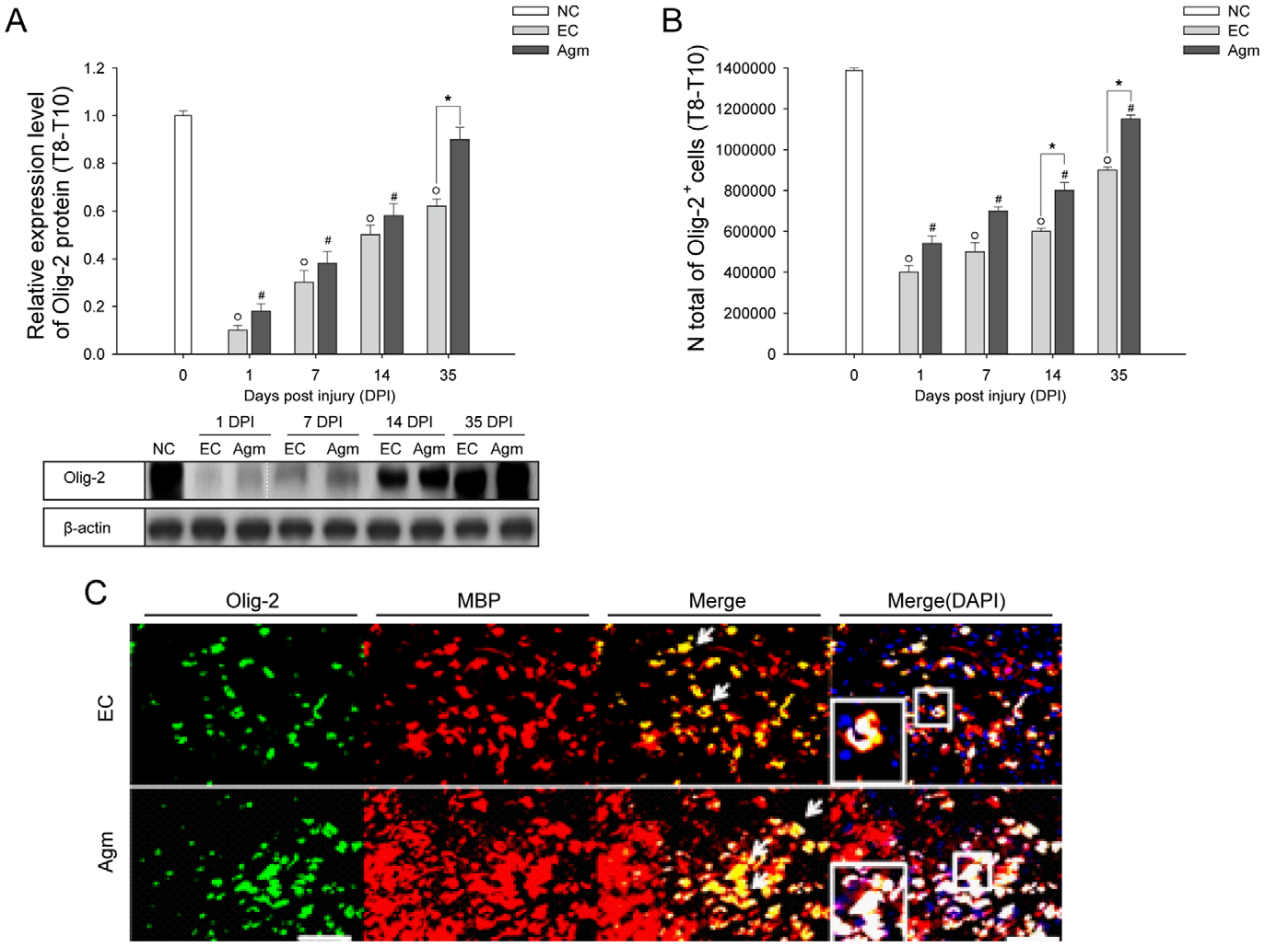
Agmatine treatment increased the expansion of oligodendrocytes following SCI. (A) Quantification of Olig-2 expression was determined by western blot analysis. The bar graph represents densitometry of the mean Olig-2 expression. Olig-2 expression was significantly higher in the Agm treated group (*n* = 5) compared with the EC group (*n* = 5) at 35 DPI. (B) Olig-2^+^ cell number were acquired using CAST analysis in the Th 8 - Th 10 segments of the injured spinal cord. The number of Olig-2^+^ cells in the Agm treated group (*n* = 5) were significantly increased compared to EC group (*n* = 5) at 14 and 35 DPI. (C) The spinal cords were immunostained with Olig-2 (green) and MBP (red) antibodies at 35 DPI. The number of Olig-2^+^/MBP^+^ cells were higher in the Agm treated group (*n* = 5) than in the EC group (*n* = 5) around the lesion site at 35 DPI. Higher magnification images revealed intact myelin rings surrounding the axons in Agm treated group, whereas disrupted myelin was found in EC group (see figure in the box). Scale bars: 50 µm. †, *p*<0.05 NC group vs EC group; #, *p*<0.05 NC group vs Agm treated group; *, *p*<0.05 EC group vs Agm treated group. Results represent mean ± S.E.M.

These remyelination effects were further confirmed by checking the Olig-2 (oligodendrocyte marker) expression by western blotting. Western blot analysis showed that the Olig-2 protein expression in Agm treated group (*n* = 5) was increased at all the time periods (from 1 DPI to 35 DPI) after compression SCI compared with that of the EC group (*n* = 5) and the values reached significance at 35 DPI (Fig. 3A). Furthermore the number of Olig-2^+^ cells were counted in the EC group (*n* = 5) and Agm treated group (*n* = 5) using CAST analysis program. The CAST counting results showed that the numbers of Olig-2^+^ cells were higher in the total spinal cord (Th 8 - Th 10 segments) at 1, 7, 14 and 35 DPI and significant increase was recorded at 14 and 35 DPI (Fig. 3B). There was an increase in the intensification of Olig-2^+^ cells in the rostral (Th 8), the lesion (Th 9), and the caudal segments (Th 10) of the injured spinal cord in the Agm treated group at 1, 7, 14, and 35 DPI and the significant difference between the groups (Agm treated group vs EC group) were recorded at 35 DPI (Fig. S4). Furthermore, remyelination (within the perimeter of the lesion site) in the injured spinal cord was confirmed by double immunostaining with Olig-2 (green) and MBP (red) at 35 DPI (Fig. 3C). Confocal microscopy results showed higher intensity of Olig-2^+^/MBP^+^ cells (rings formation indicated by a white arrow in Fig. 3C) in the Agm treated group (*n* = 5) compared with the EC group (*n* = 5). Considering that endogenous oligodendrocyte progenitor cells (NG2^+^) local to the lesion site are to be the source of new myelinating cells, experiments were done to check the expression of NG2^+^ cells in Olig-2^+^and GFAP^+^ cell population at 7 and 35 DPI. Our immunohistochemical staining results showed that the expansion of NG2^+^/Olig-2^+^ cells outnumbered in the Agm treatment group both at 7 days and 35 DPI compared to EC group. But the NG2^+^/GFAP^+^ cells were less in Agm treated group both at 7 and 35 days DPI compared to EC group (Fig. S3).

These results depict that Agm treatment could promote the formation of myelin sheath and might facilitate the remyelination process following SCI.

### Agmatine Treatment Protects the Damaged Neurons Following SCI

**Figure 4:**
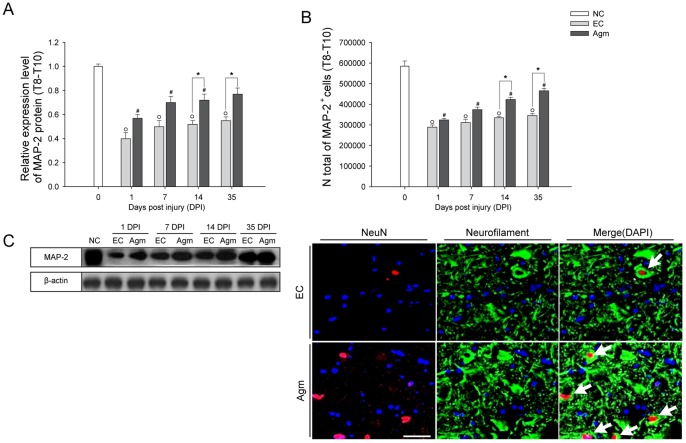
Agmatine treatment prevented neuronal cell loss following SCI. (A) Western blots of MAP-2 protein expression at 1, 7, 14, and 35 DPI. The representative graphs showed that the MAP-2 expression in Agm treated group (*n* = 5) was increased from 1 to 35 DPI and the increase was significant at 14 and 35 DPI compared to EC group (*n* = 5). (B) Stereological count of MAP-2^+^ cells. The number of MAP-2^+^ cells were increased in the Agm treated group (*n* = 5) at 1, 7, 14 and 35 DPI compared with the EC group (*n* = 5) in Th 8 - Th 10 segments of the spinal cord. (C) The injured spinal cord tissues were immunostained with NeuN (red) and Neurofilament (green). Results showed higher number of NeuN^+^/Neurofilament^+^ cells in the Agm treated group (*n* = 5) compared with the EC group (*n* = 5) at 35 DPI. Scale bars: 50 µm. †, *p*<0.05 NC group vs EC group; #, *p*<0.05 NC group vs Agm treated group; *, *p*<0.05 EC group vs Agm treated group. Results represent mean ± S.E.M.

Approaches to treat SCI include prevention of damaged neurons and regeneration of tissue loss. Strategies aimed to prevent neuronal damage will arise from secondary injury processes providing some hope for tissue sparing and improved functional outcome [37]. To ascertain this, quantitative measurement of MAP-2 protein expression was done using western blot analysis after SCI. The densitometry results showed that the MAP-2 expression in the Agm treated group (*n* = 5) was increased at 1, 7, 14 and 35 DPI (*n* = 5) and the values reached significance at 14 and 35 DPI compared with the EC group (Fig. 4A). CAST analysis showed that the expansion of total number of surviving neurons (immunostained with MAP-2 antibody) in the total spinal cord (Th 8–Th 10 segments) (Fig. 4B) and in the rostral (Th 8), the lesion (Th 9) and the caudal (Th 10) segments of the spinal cord were increased in Agm treated group at all the time intervals compared with EC group (*n = *15, per group) (Fig. S5) and the increase was significant at 14 and 35 DPI compared with the EC group in the total spinal cord and in the rostral and caudal segments (*n* = 15, per group). These results suggest that Agm treatment rescue the damaged neurons and accelerate the regeneration of damaged neurons after SCI. The neuronal nucleus (NeuN) and neurofilament (NF) expressions were found almost exclusively in neuronal cells and support the cytoskeleton following SCI. In our study immunofluorescence staining was done to detect the expressions of NeuN^+^/NF^+^ cells in EC and Agm treated groups. Immunofluorescence results showed higher number of NeuN^+^/NF^+^ cells in Agm treated group (*n* = 5) compared with the EC group (*n* = 5) and it seems that Agm treatment preserved the formation of dendrites and cell bodies of neurons around the lesion site in the injured spinal cord at 35 DPI (Fig. 4C).

These findings suggest that Agm treatment attenuate the neuronal damage and aid for the neuronal survival following SCI.

### Agmatine Treatment Attenuated Glial Scar Formation and Reactive Gliosis at the Injury Site Following SCI

SCI often results in permanent neurological impairment and axonal regeneration is made difficult due to astrocytes activation, oxidative stress, inflammation, cell death, and axon disruption [38]. Recently it was reported that Agm treatment could support neuroregeneration by reducing the collagen scar area by decreasing the expression of TGFß-2 and increasing the expression of BMP-7 following SCI [17], [23].

**Figure 5:**
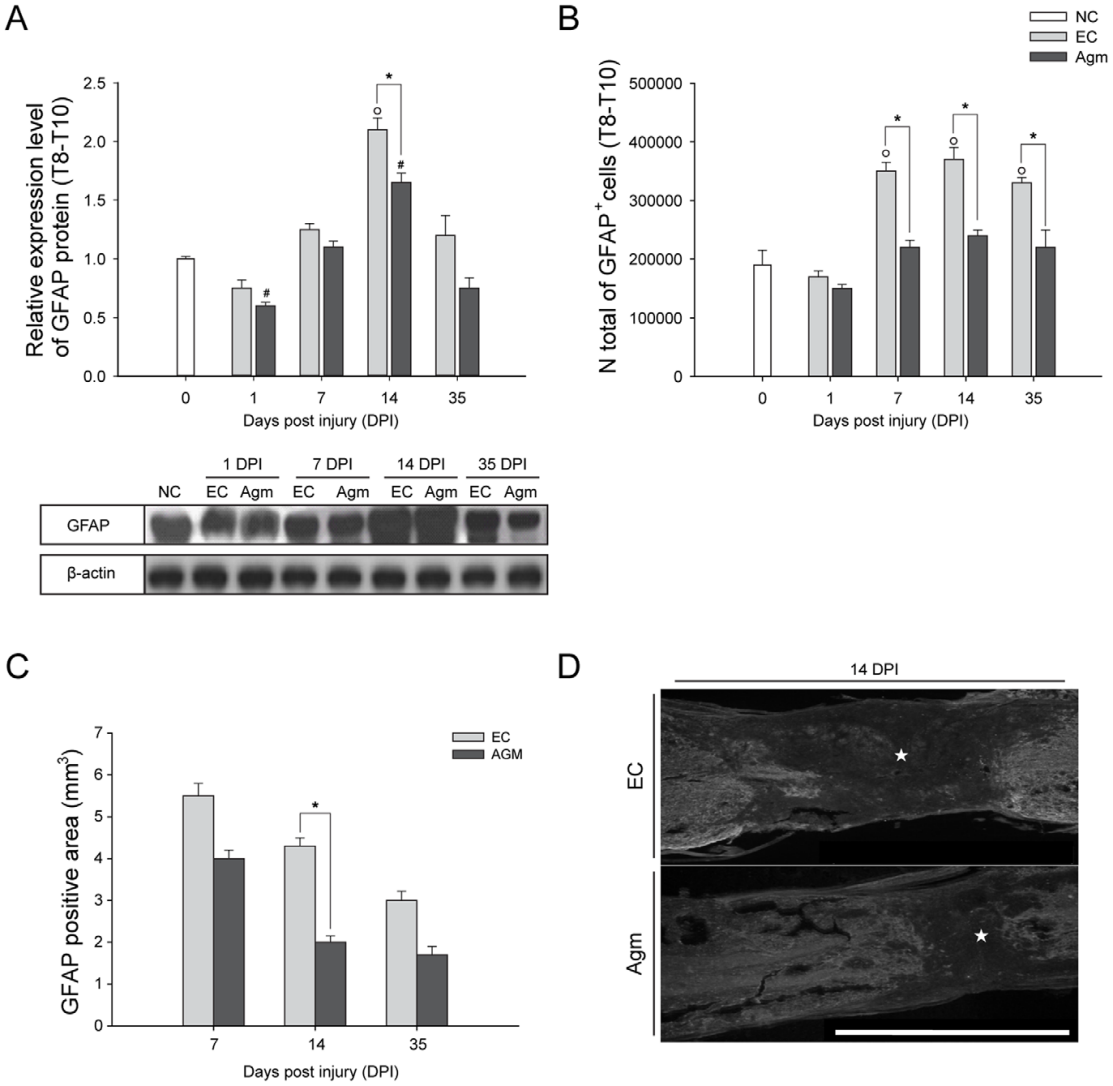
Agmatine treatment reduced the gliosis activation following SCI. (A) Representative western blots of GFAP expression at each time point in the EC and Agm treated group along with β-actin as a loading control**.** The GFAP expressions were substantially decreased after SCI in the Agm treated group (*n* = 5) compared with the EC group (*n* = 5). (B) The total numbers of GFAP^+^ cells in the injured spinal cord (Th8– Th10 segments) were counted by CAST analysis. Results showed a significant reduction of GFAP^+^ cells in the Agm treated group (*n* = 5) at 7, 14, and 35 DPI compared with the EC group (*n* = 5). (C) GFAP positive area (glial scar area) was measured at 7, 14, and 35 DPI using an image analysis program. The Agm treated (*n* = 4) group showed reduction in the glial scar formation area at all the time periods and the reduction was found to be statistically significant at 14 and 35 DPI compared to EC group (*n* = 4). (D) The EC (*n* = 4) and Agm treated group (*n* = 4) were immunostained with GFAP antibody to measure the glial scar area following SCI at 14 DPI. The GFAP positive area was decreased after SCI in the Agm treated group compared with the EC group (★, indicate the glial scar area). Scale bars: 2.0 mm. †, *p*<0.05 NC group vs EC group; #, *p*<0.05 NC group vs Agm treated group; *, *p*<0.05 EC group vs Agm treated group. Results represent mean ± S.E.M.

In our study, the western blot results demonstrated significant decrease in the GFAP protein expression in the Agm treated group (*n* = 5) at all the time periods (1, 7, 14, and 35 DPI) and the decrease was statistically significant at 14 DPI (Fig. 5A) compared with EC group (*n* = 5). The number of GFAP^+^ cells were counted using CAST analysis from total spinal cord (Th 8–Th 10 segments) and at the rostral (Th 8), the lesion (Th 9), and the caudal (Th 10) segments of the spinal cord in EC and Agm treated groups (*n* = 5, per group). The CAST analysis revealed significant decrease in the total number of GFAP^+^ cells in the total spinal cord and also in the rostral, lesion and caudal segments of the spinal cord in Agm treated group compared with the EC group at 7, 14, and 35 DPI. (Fig. 5B, S6). And also, the glial scar formation after SCI was determined by GFAP immunoreactivity at the injured site. The GFAP immunoreactivity was measured using the image analysis program. The results showed that Agm treatment significantly decreased the GFAP immunopositive area (*n* = 5) compared with the EC group (*n* = 5) at 7, 14 and 35 DPI and the decrease was found to be statistically significant at 14 and 35 DPI (Fig. 5C) suggesting that Agm treatment significantly attenuated the glial scar formation at 14 days following SCI (★, indicate glial scar area Fig. 5D).

### Agmatine Treatment Prevented the Neuronal & Oligodendrocytes Cell Loss and Attenuated the Astrocytes Formation Around the Lesion Site Following SCI

Earlier it was reported that in the intact adult spinal cord the glial progenitor cells occupy 80% of the total cells. However, there is a substantial net increase in the progeny of damaged ependymal and astrocytes lineage cells following SCI [39], [40]. Our present study first aimed to count the total cell numbers of surviving neurons, astrocytes and oligodendrocytes in the NC, EC and Agm treated groups (*n = *5, per group) in Th 8–Th 10 segments of the spinal cord using CAST analysis program (cells were read as actual number;×10^6^ cells/mm^3^) at 1, 7, 14 and 35 DPI using MAP-2, GFAP and Olig-2 antibodies. The CAST results showed that in the control mice (NC group) the average cell number representing neurons were 5.742±0.274 (×10^6^ cells/mm^3^). After SCI the total neuronal cell numbers were decreased in the EC group. However, Agm treatment dramatically attenuated the total neuronal cell loss following SCI and the average cell count number were significantly higher at all the time points compared with the EC group and the average numbers compared with EC group (EC vs Agm) were: 2.875±0.043 vs 3.268±0.065 at 1 day, 3.127±0.065 vs 3.745±0.056 at 7 days, 3.359±0.095 vs 4.251±0.109 at 14 days and 3.517±0.144 vs 4.622±0.273 (×10^6^ cells/mm^3^) at 35 DPI. Specifically, the average numbers of surviving neurons in the Agm treated group were recorded to be almost similar to that NC group at 35 DPI.

The total number of oligodendrocytes were also counted using CAST analysis. The CAST counting results revealed that the average Olig-2^+^ cell numbers in the NC group were 14.078±2.037 (×10^6^ cells/mm^3^). SCI resulted in the significant loss of oligodendrocytes. But, and the average cell count number from the CAST results showed 4.018±0.030, 4.997±0.205, 5.993±0.082 and 9.018±0.206 (×10^6^ cells/mm^3^) at 1, 7, 14 and 35 DPI respectively in EC group. But, Agm treatment prevented the oligodendrocytes cell loss and the average Olig-2^+^ cells were 5.404±0.008, 6.964±0.04, 8.030±0.112 & 12.004±0.431 (×10^6^ cells/mm^3^) at 1, 7, 14 and 35 DPI and the average cell numbers were found to be significant at 7 and 14 days compared to the EC group.

The total numbers of GFAP^+^ cells were counted in the NC, EC and Agm treated groups. CAST analysis showed that in the NC group the average total cell numbers of GFAP^+^ cells were 2.092±0.281 (×10^6^ cells/mm^3^). Following SCI the total number of GFAP^+^ cells were decreased in the Agm treated group compared to the EC group and the decrease between EC vs Agm were: 1.702±0.007 vs 1.502±0.005 at 1 day, 3.502±0.171 vs 2.205±0.108 at 7 days, 3.701±0.148 vs 2.395±0.086 at 14 days and 3.752±0.086 vs 2.503±0.055 (×10^6^ cells/mm^3^) at 35 DPI.

The overall CAST results suggest that Agm treatment significantly prevented the neurons and oligodendrocytes cell loss and inhibited the formation of astrocytes. Moreover, the average cell numbers of all the cell types (neurons, oligodendrocytes and astrocytes) in Agm treated group at 35 DPI were almost reached to the normal control group (Table 1).

**Table 1:** The average numbers of neurons and glial cells in injured spinal cord in Th 8–Th 10 segments following SCI.

		EC	Agm	NC
**Neurons**	**1 DPI**	2.875±0.043†	3.268±0.065^#^	5.742±0.274
	**7 DPI**	3.127±0.065†	3.745±0.056^#^	5.742±0.274
	**14 DPI**	3.359±0.095†	4.251±0.109^#,^*	5.742±0.274
	**35 DPI**	3.517±0.144†	4.622±0.273^#^ *	5.742±0.274
**Oilgo** **dendrocytes**	**1 DPI**	4.018±0.030†	5.404±0.008^#^	14.078±2.037
	**7 DPI**	4.997±0.205†	6.964±0.043^#^	14.078±2.037
	**14 DPI**	5.993±0.082†	8.030±0.112^#,^*	14.078±2.037
	**35 DPI**	9.018±0.206†	12.004±0.431^#,^*	14.078±2.037
**Astrocytes**	**1 DPI**	1.702±0.007	1.502±0.005	2.092±0.281
	**7 DPI**	3.502±0.171†	2.205±0.108*	2.092±0.281
	**14 DPI**	3.701±0.148†	2.395±0.086*	2.092±0.281
	**35 DPI**	3.752±0.086†	2.503±0.055*	2.092±0.281

For the computer assisted stereological toolbox (CAST) analysis the spinal cord containing Th 8–Th 10 segments were chosen for the study. The table showing the distribution of neurons, oligodendrocytes and astrocytes in NC group (*n* = 5), EC group (*n* = 5) and Agm treated group (*n* = 5) at 1, 7, 14, and 35 DPI. The cells were measured per mm^3^ (Units:×10^6^ cells/mm^3^). The Agm treated group showed an overall increase of the total number of cells with high proportions of MAP-2^+^ and Olig-2^+^ cells and less GFAP^+^ cells compared with the EC group at all the time periods following SCI. †, *p*<0.05 NC group vs EC group; #, *p*<0.05 NC group vs Agm treated group;*, *p*<0.05 EC group vs Agm treated group. Results represent mean ± S.E.M.

Data are means ± S.E.M.

† , *P*<0.05 for NC group vs EC group; #, *P*< 0.05 for NC group vs Agm group; *, *P*<0.05 for EC vs Agm group.

NC: normal control group; EC: experimental control group; Agm: agmatine treated group; DPI: days post injury.

Cell number:×10^6^ cells/mm^3^; *n* = 5 per group.

### Agmatine Treatment Modulated the BMP-2/4/7 Expressions Following SCI

Bone morphogenetic proteins (BMPs) play a critical role in regulating cell fate determination during central nervous system (CNS) development and BMP- 2/4/7 expressions in particular modulates cell differentiation at the injury site following SCI [16]. Taking into consideration the important roles of BMP- 2/4/7 expressions following SCI, here, we intended to investigate whether Agm treatment could modulate the BMP- 2/4/7 protein expressions and contribute for neurological recovery following SCI.

**Figure 6:**
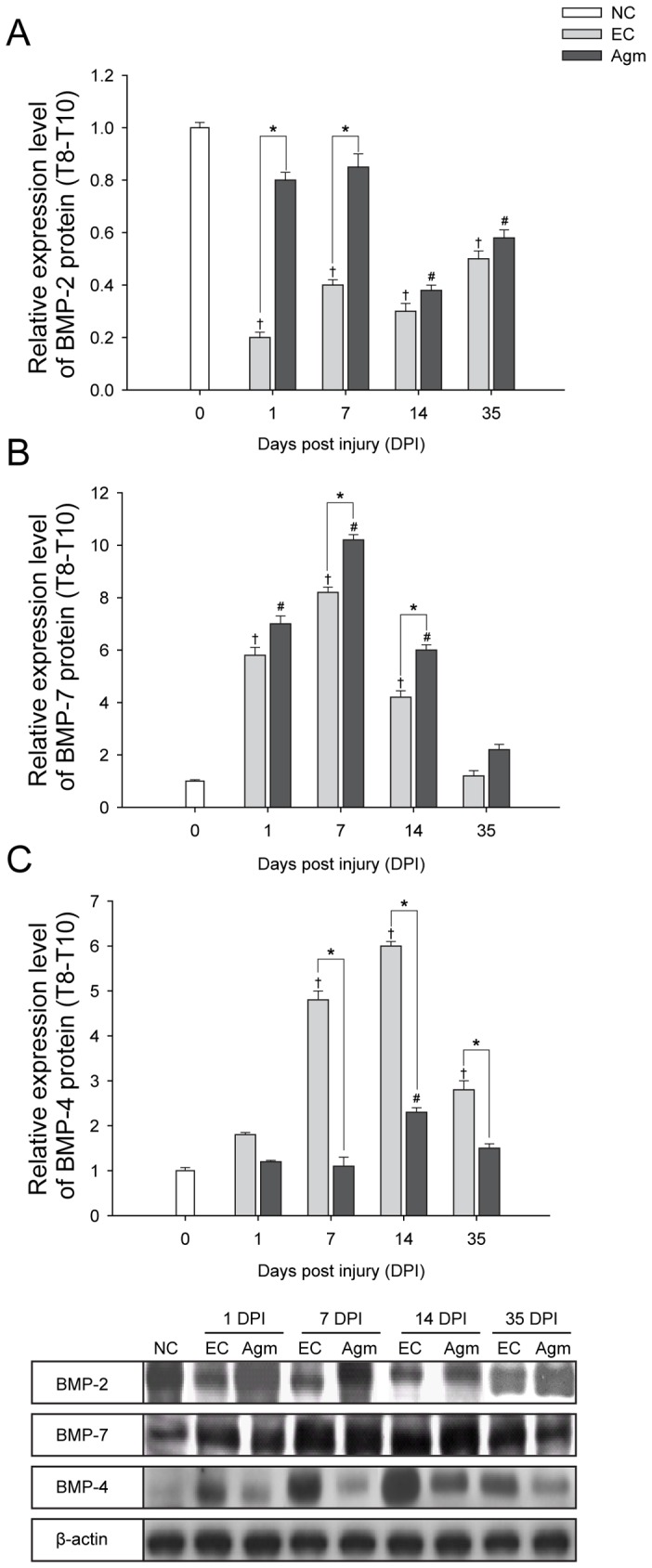
Agmatine treatment modulated the BMP- 2/4/7 expressions after SCI. Quantification of BMP- 2/4/7 protein expressions was determined by western blot analysis. The bar graph represents densitometry measurement of BMP- 2/4/7 protein expressions. (A) BMP- 2 protein expression was significantly higher in the Agm treated group (*n* = 5) compared with the EC group (*n* = 5) at 1 and 7 DPI. (B) BMP- 7 protein expression was higher in the Agm treated group (*n* = 5) compared with the EC group (*n* = 5) at 1, 7, 14, and 35 DPI and the increase was significant at 7 & 14 DPI. (C) BMP- 4 expression was significantly lower in the Agm treated group (*n* = 5) compared with the EC group (*n* = 5) at 7, 14 & 35 DPI following SCI. †, *p*<0.05 NC group vs EC group; #, *p*<0.05 NC group vs Agm treated group; *, *p*<0.05 EC group vs Agm treated group. Results represent mean ± S.E.M.

The quantitative western blot results showed that the BMP- 2/7 protein expressions were increased at 1, 7, 14, and 35 DPI and the values reached significant at 1 & 7 DPI and 7 & 14 DPI respectively in Agm treated group (*n* = 5) compared with the EC group (*n* = 5) (Fig. 6A, 6B). Conversely, the quantitative results of the BMP- 4 protein expression was decreased at 1, 7, 14, and 35 DPI and the values were significant at 7, 14 and 35 DPI in the Agm treated group (*n* = 5) compared with those of the EC group (*n* = 5) (Fig. 6C).

### Agmatine Treatment Modulates the Expansion of Oligodendrocytes Progenitor Cells (NG2^+^) via BMP- 2/4/7 Expressions Following SCI

Endogenous oligodendrocyte progenitor cells (NG2) local to the lesion site differentiate into oligodendrocytes and are responsible for myelin repair [41], [42]. The expression pattern of NG2^+^ in BMP- 2/4/7^+^ cell populations was determined by immunofluorescence staining. The results suggested that the NG2^+^/BMP- 2^+^ cells were higher in the Agm treated group compared with EC group at 7 and 35 DPI. However, the NG2^+^/BMP- 4^+^ cell population were decreased at 7 DPI and the NG2^+^/BMP- 4^+^ cells were almost disappeared in the Agm group at 35 DPI. Conversely the expansion of NG2^+^/BMP- 7^+^ cells was increased around the lesion site in Agm treated group at 35 DPI compared to EC group (*n* = 5) (Fig. S8).

### Agmatine Treatment Increased the BMP- 2/7 Expressions in Neurons and Oligodendrocytes and Decreased the BMP- 4 Expressions in Astrocytes and Oligodendrocytes Following SCI

BMPs are known to regulate proliferation or differentiation of neurons, oligodendrocytes, and astrocytes during CNS development [43]. We hypothesized whether Agm treatment could modulate BMPs expression in neurons, astrocytes and oligodendrocytes after SCI. Recent findings demonstrated that BMPs show potential relationship with neurons and glial cells in the normal/injured spinal cord [16]. In this study immunofluorescence and DAB staining (CAST analysis) were performed to co-localize and count the BMP- 2/4/7^+^ cells in neurons, oligodendrocytes and astrocytes at 1, 7, 14 and 35 DPI.

**Figure 7:**
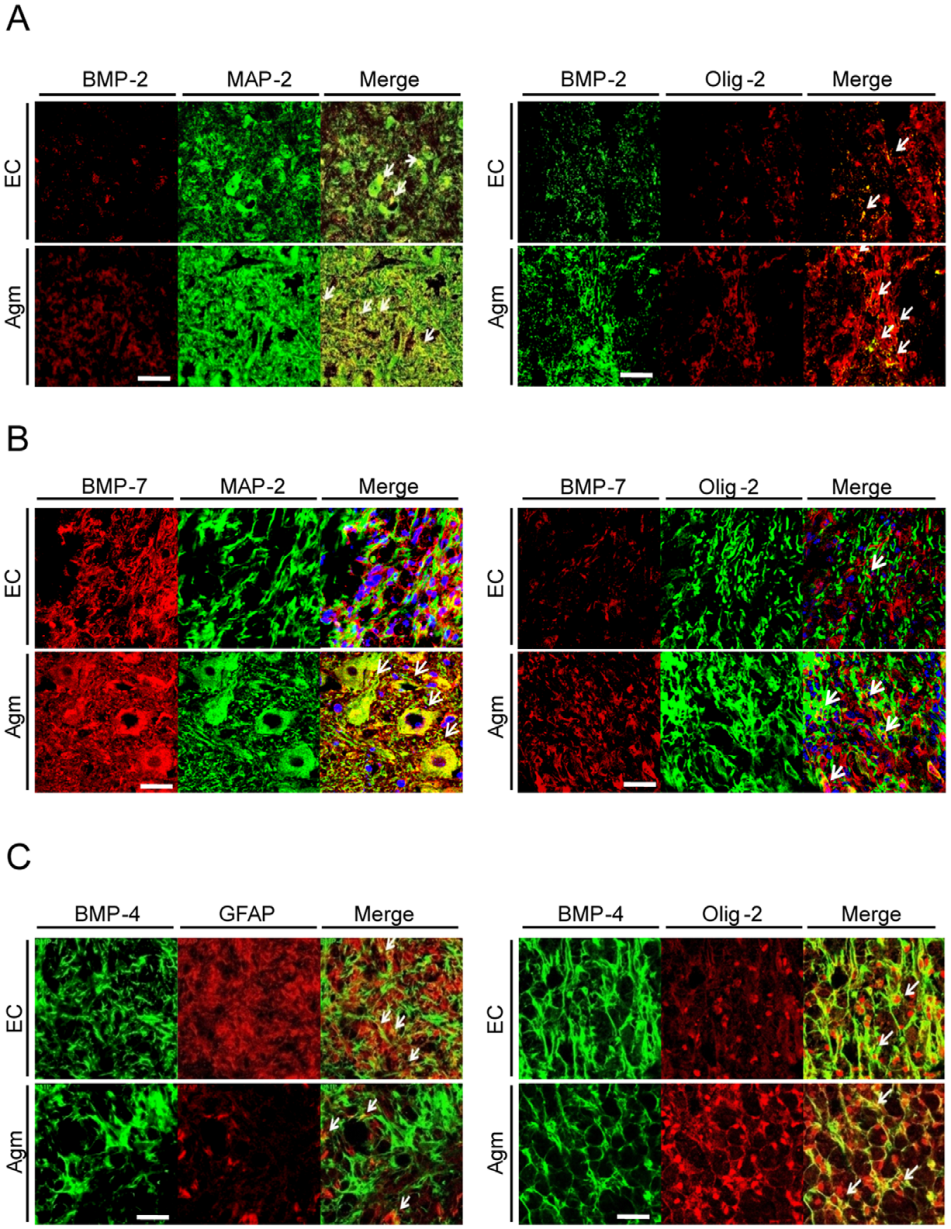
Confocal microscopic images of BMP- 2/4/7 expressions in neurons, oligodendrocytes and astrocytes after agmatine treatment following SCI. (A) Dual immunofluorescence was done to localize the BMP- 2 expression in neurons (MAP-2) and oligodendrocytes (Olig-2) after SCI. The BMP- 2^+^/MAP- 2^+^& BMP- 2^+^/Olig-2^+^ cells around the lesion site were higher in the Agm treated group (*n* = 4) compared with the EC group (*n* = 4) at7 DPI. Scale bars: 50 µm. (B) The immunostaining of BMP- 7 (red) with MAP-2 (green) showed increased number of BMP- 7^+^/MAP-2^+^ cells in the Agm treated group (*n* = 5) when compared with the EC group (*n* = 5) at 7 days after SCI. The immunostaining of BMP- 7 with Olig-2 showed an increased number of BMP- 7^+^/Olig-2^+^ cells in the Agm treated group (*n* = 5) compared to EC group (*n* = 5) at 14 DPI. Scale bars: 10 µm. (C) The immuno co-localization of BMP- 4^+^ cells in astrocytes (GFAP) showed reduced number of BMP- 4^+^/GFAP^+^ cellsaround the lesion site in the Agm treated group (*n* = 4) compared with the EC group (*n* = 4) at7 DPI. Whereas, BMP- 4^+^/Olig-2^+^ cells were higher in the Agm treated group compared with EC at 35 DPI. The scale bars: 10 µm.

**Figure 8:**
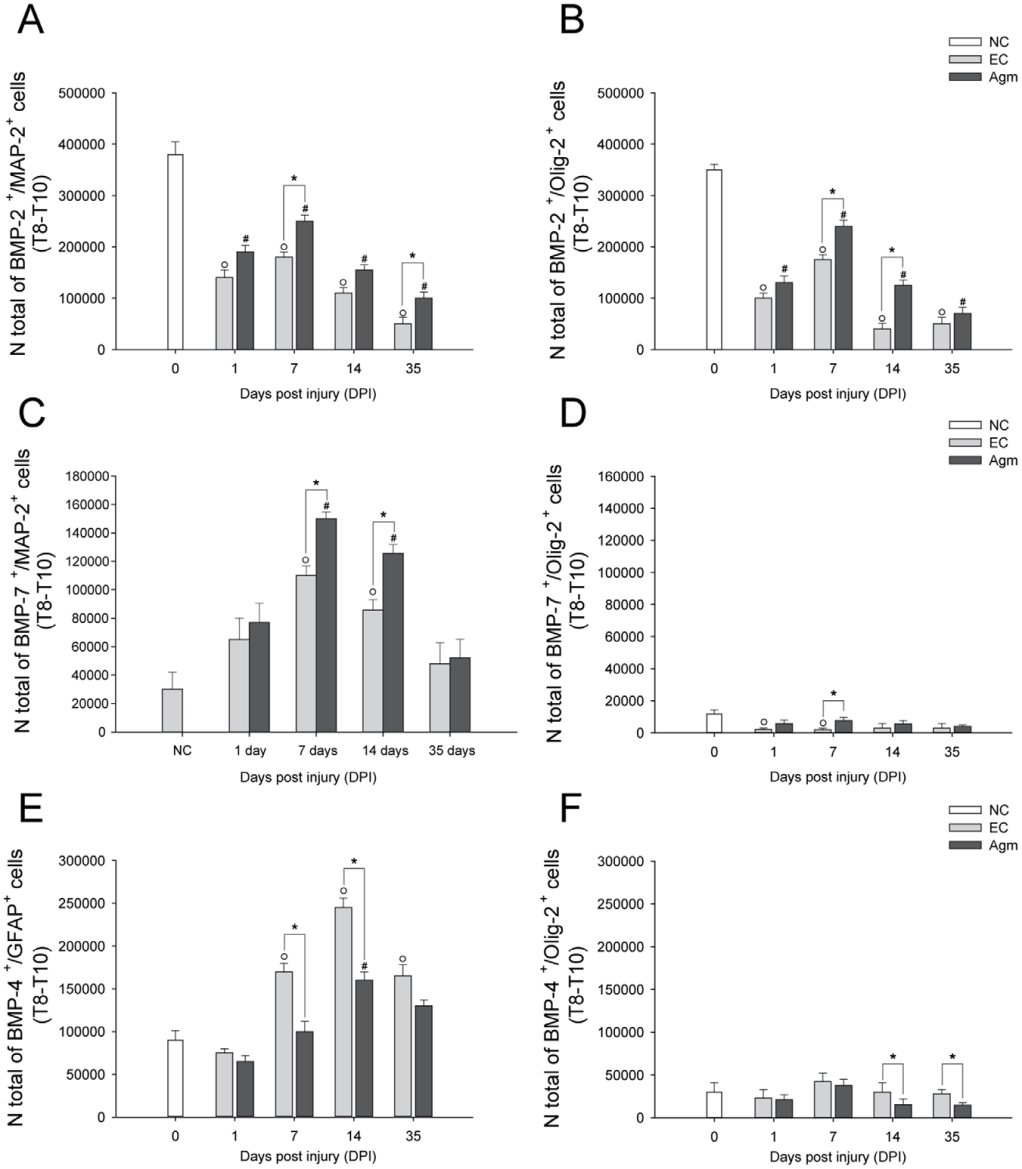
CAST analysis showed that agmatine treatment modulated BMP- 2/7 expressions in neurons & oligodendrocytes and BMP- 4 expressions in astrocytes & oligodendrocytes following SCI. (A) Stereological counting of BMP- 2^+^/MAP-2^+^and (B) BMP- 2^+^/Olig-2^+^ cells in the injured spinal cord (Th 8–Th 10 segments). The number of BMP- 2^+^/MAP-2^+^ and BMP- 2^+^/Olig-2^+^ cells were significantly increased in the Agm treated group (*n* = 5) compared with the EC group (*n* = 5) at 7 & 35 DPI and 7 & 14 DPI (C) BMP- 7^+^/MAP-2^+^ and (D) BMP- 7^+^/Olig-2^+^ cells in the Th 8–Th 10 segments of the injured spinal cord. The number of BMP-7^+^/MAP-2^+^ and BMP-7^+^/Olig-2^+^ cells were significantly increased in the Agm treated group (*n* = 5) compared with the EC group (*n* = 5) at 7 & 14 days and 7 days respectively following SCI. (E) BMP- 4^+^/GFAP^+^ and (F) BMP- 4^+^/Olig-2^+^ cells in Th 8–Th 10 segments of the injured spinal cord. The number of BMP- 4^+^/GFAP^+^ and BMP- 4^+^/Olig-2^+^ cells were decreased at all the time periods in the Agm treated group (*n* = 5) compared with the EC group (*n* = 5) and the decrease was significant at 7 & 14 days and 14 & 35 days respectively following SCI. †, *p*<0.05 NC group vs EC group; #, *p*<0.05 NC group vs Agm treated group; *, *p*<0.05 EC group vs Agm treated group. Results represent mean ± S.E.M.

The immunofluorescence staining results showed higher number of BMP- 2^+^/MAP-2^+^ & BMP- 2^+^/Olig-2^+^ cells in Agm treated group compared to the EC group at 7 and 14 days respectively following SCI (Fig. 7A). Similarly, CAST results showed the total number of BMP- 2^+^/MAP-2^+^ and BMP- 2^+^/Olig-2^+^ cells were higher in the Agm treated group (*n* = 5) compared with the EC group (*n* = 5) at 1, 7, 14 and 35 DPI and the increase in numbers were significant at 7 & 35 DPI and 7 & 14 DPI respectively in the Agm treated group (*n* = 5) compared with EC group (*n* = 5) (Fig. 8A, 8B). The average cell numbers representing the BMP- 2 co-localized cells with MAP-2 and Olig-2 expressions were provided in the Table S2.

It was reported that BMP- 7 has been shown to exert neuroprotective effect after traumatic SCI and promote the functional recovery after contusion SCI [17]. Here, we investigated whether Agm could modulate the BMP- 7 expressions in neurons and oligodendrocytes after SCI. Dual immunofluorescence staining was performed to localize the BMP- 7^+^/MAP-2^+^ and BMP- 7^+^/Olig-2^+^ cells following SCI. The immunofluorescence staining results showed increased number of BMP- 7^+^/MAP-2^+^ and BMP- 7^+^/Olig-2^+^ cells at 7 and 14 DPI in the Agm treated group (*n* = 4) compared with the respective EC group (*n* = 4) (Fig. 7B). DAB immunostaining was also performed to localize the BMP- 7 expression in neurons and oligodendrocytes and the total number of BMP- 7^+^/MAP-2^+^ and BMP- 7^+^/Olig-2^+^ cells were counted using the CAST analysis program. The CAST data showed that the number of BMP- 7^+^/MAP-2^+^ and BMP- 7^+^/Olig-2^+^ cells were increased in the Agm treated group (*n* = 5) compared with the EC group (*n* = 5) at 1, 7, 14, and 35 DPI and the values were significant at 7 & 14 DPI and 7 DPI respectively compared with that of the EC group (Fig. 8C, 8D). The average cell numbers representing the BMP-7 co-localized cells with MAP-2 and Olig-2 expressions was provided in the Table S2.

BMP- 4 can take part in inhibiting oligodendrocytes specification and differentiation to promote astrocytes proliferation after injury [16]. Previous findings suggest that BMP- 4 increases reactive gliosis and glial scar formation at the lesion site following SCI [44], [45]. To ascertain the involvement of BMP- 4 expression in modulating gliosis, immmunofluorescence staining was performed to localize the BMP- 4^+^ cells in astrocytes and oligodendrocytes. Results showed that Agm treatment decreased the BMP- 4^+^/GFAP^+^ cell population at 7 DPI and increased the BMP- 4^+^/Olig-2^+^ cells expansion at 35 DPI (Fig. 7C). However, BMP- 4 expression was not found to be co-localized with MAP-2 both in the EC and the Agm treated group (Fig. S7).

Simultaneously, CAST analysis was performed to count the total number of BMP- 4^+^ cells in astrocytes and oligodendrocytes in the EC (*n* = 5) and Agm treated group (*n* = 5) at 1, 7, 14, and 35 DPI. The CAST results showed that the total numbers of BMP- 4^+^/GFAP^+^ and BMP- 4^+^/Olig-2^+^ cells in the Agm treated group were decreased at all the time periods compared with the EC group and the decrease was found to be statistically significant at 7 & 14 DPI in astrocytes and at 14 & 35 DPI in oligodendrocytes (Fig. 8E, 8F) compared with EC group. The average cell numbers representing the BMP- 4 co-localized cells with GFAP and Olig-2 expressions was provided in Table S2.

## Discussion

Agmatine (Agm), an N-methyl-D-aspartate receptor (NMDAR) antagonist and nitric oxide synthase (NOS) inhibitor prevent adaptive changes in neuronal function and selectively relieves allodynic, hyperalgesic, and autotomy-like states accompanying spinal cord injury (SCI) [10]. In neurons of the brain and spinal cord, Agm is packaged in synaptic vesicles and released upon neuronal depolarization. The evidence of neuromodulatory role for Agm is shown to antagonize NMDAR [46]–[48].

In the present study, Agm treatment (1) improved locomotory and physiological functions, (2) facilitated axonal remyelination, (3) promoted protection of neurons, (4) attenuated glial scar formation, and (5) modulated the BMP- 2/4/7 expressions in neuronal and glial cells which might be critical factor for the improvements of the impairments following SCI. Previous studies suggested that mice and rat recovered from locomotor dysfunctions after Agm treatment in contusion and transaction SCI models [23], [49]. Corroborating with the earlier studies our funtional recovery test results also demonstrated that Agm treated mice significantly regained the ability to make weight-supported steps with their hind limbs, which was in stark contrast to EC group (saline treated mice) that only regained the ability to make rhythmic sweeping motions with their hind limbs after SCI. SCI leads to bladder and renal dysfunctions resulting in failure of self urination and ultimately delay the overall recovery process [50]. In our study, injury induced bladder dysfunction was restored with Agm treatment is of particular clinical relevance for quality of life, since restoration of bladder function is consistently ranked as a higher priority than walking [30]. SCI is a devastating disease and the resultant pathology arises from both primary and secondary injury mechanisms resulting in inflammation and oxidative stress leading to low survival rate of the oligodendrocytes [51]. In general oligodendrocytes provide myelin sheath formation for enhanced axonal transmission [52], [53]. But, SCI results in the loss of myelin forming cells which leads to exposure of surviving axons, impairs their conductive capacity and limits the recovery of neural function [54]. Oligodendrocytes that survive the demyelinating insult are not thought to be able to contribute to remyelination [55], [56]. Instead endogenous oligodendrocyte progenitor cells, NG2^+^ cells, local to the lesion are believed to be the source of new myelinating cells and there are evidences that these cells are responsible for myelin repair [57]–[59]. Our transmission electron microscopic (TEM) studies suggested that Agm treatment showed better pattern of myelination in the lesioned cords compared to EC group and luxol fast blue staining indicated the reconstruction of lost myelin and neurons. Immunostaining results suggested that Agm treatment increased the intensification of oligodendrocyte progenitor cells (NG2^+^) associated with mature oligodendrocytes (Olig-2^+^) with less expansion of NG2^+^/GFAP^+^ cells in Agm treated group compared to EC group following SCI. These events of remyelination were further confirmed by quantification of Olig-2 expression by western blotting and CAST analysis. The Olig-2^+^/MBP^+^ cells were increased in the Agm treated group compared to those in the EC group after SCI, suggesting that Agm treatment might boost the regeneration of damaged oligodendrocytes, prevent myelin loss, and assist in enhancing axonal remyelination following SCI.

In the spinal cord, neurons coordinate rhythmic locomotor movements [60], [61] and these spinal neurons require neuromodulators like serotonin (5-hydroxytryptamine or 5-HT) to function, setting them into a state of readiness for sensiromotor integration [62], [63]. The neuromodulator, 5-HT is one of the important endogenous monoamine synthesized by specialized neurons in the brainstem (ie, raphe nucleus and parapyramidal region) with extended projections throughout the brain as well as in the spinal cord as far as into the lumbar and sacral segments [1]. During the chronic phase of SCI, drugs that activate 5-HT may contribute for regaining the sensiromotor integration showing the strong correlation between locomotor recovery and lumbosacral spinal 5-HT status after SCI [64], [65]. Our immunostaining results with 5-HT antibody (represents serotogenic fiber staining) showed increase in the caudal 5-HT fiber density in the Agm treated group and the morphology of the serotogenic fibers were almost similar compared to normal control mice. However the serotogenic fibers showed broken morphology in the EC group and were appeared like beads both at 14 and 35 DPI. These results depicts that local sprouting of the serotogenic fibers could accout for the improvement of functional recovery in the Agm treated mice.

SCI causes neuronal and glial cells loss resulting in motor neuron dysfunctions. SCI repair focuses on finding ways to improve the axonal re-growth, the axonal re-connection and replace or protect damaged neurons and glial cells [66], [67]. Given that Agm has been reported to possess neuroprotective effects, our western blot and immunostaining data demonstrated that Agm treatment increased the MAP-2, NeuN, and NF expressions following SCI. Parallel to our western blot and immunostaining results, CAST analysis also revealed an increased expansion of MAP-2^+^ cell population in the Agm treated group. The p53 associated apoptosis is considered to be one of the common mechanisms of cell death in neurodegenerative diseases. Our immunohistochemical staining results revealed fewer number of p53 positive cells in the Agm treated group compared with EC group both at 14 and 35 DPI. The above findings suggest that Agm treatment rescued the neuronal cell loss and exerted neuroprotection in the damaged cords.

Following SCI one of the physical barriers for regenerating axons is glial scar formation, which consists predominately of reactive astrocytes. Axons cannot regenerate beyond the glial scar, and they take a dystrophic appearance of stalled growth [51] and GFAP is one of the major intermediates reported to be localized in the cytoplasm responsible for the surrounding astrocytes population [68]. Finding means of inhibiting the glial scar formation offers a potential strategy for facilitating axonal regeneration. Recently, it was demonstrated that Agm treatment reduced collagen scar formation in a transected SCI mice [23]. In agreement with the above findings our current study results also demonstrated that the expansion of glial scar formation area was significantly attenuated in Agm treatment compared with the EC group. The beneficial effects of Agm on functional outcome may in part be due to increased tissue preservation within the injury site (glial scar attenuation), since the amount of preserved tissue has previously been positively correlated with functional outcome [69]. Interestingly, our western blot and CAST results also showed that the expansion of GFAP^+^ cells were significantly decreased in the Agm treated group which might facilitate the reduction in the glial scar formation following SCI.

The bone morphogenic proteins (BMPs) signalling enhances axonal outgrowth and locomotory recovery following SCI [70]. Previous studies demonstrated that BMPs play key roles as neurotrophic and growth factors during CNS development [4], [71]. In addition BMPs also play important role in regulating the development of many tissues, protecting nerve cells, promoting their re-growth, and promoting a substantial improvement in locomotor function following CNS injuries [72]. Moreover, the BMPs signaling are implicated in multiple developments such as proliferation, differentiation and dendrite formation of neuronal cells [73], [74]. In light of above evidences we intended to investigate whether Agm treatment could modulate the BMPs (BMP- 2/4/7 in particular) expressions in neurons and glial cells which might promote the glial and neuronal cell survival following SCI. Among the BMPs family, BMP- 2 promotes the differentiation of neurons and dendrite growth in cultured striatum neurons [75] and the percentage of MAP-2^+^ cells were greatly increased with BMP- 2 stimulation in a mouse embryonic telencephalon culture, however, the percentage of GFAP^+^ cells were decreased [76]. Our study results showed that the BMP- 2 expression in neurons and oligodendendrocytes were increased in Agm treated group following SCI showing the role of BMP- 2 in controlling neurogenesis and oligodendrogenesis. The expression of BMP- 7 was known to increase after CNS injury in motor neurons and might regulate differentiation of glial cells from neural progenitors which induced functional recovery after CNS injury [77], [78]. In addition, the expression of BMP- 7 was reported to exert both neuroprotective and neuroregenerative effects in animals with stroke and SCI [18], [79]. In agreement to the previous findings, our results show that Agm treatment increased the BMP- 7 expression in neurons and oligodendrocytes following SCI which might be one of the critical factor for restoration of damaged neurons and oligodendrocytes. It was reported that the expression of BMP- 4 enhanced the astrocytic lineage during CNS development and contributed to the formation of glial scar in injured spinal cord [45], [80]. BMP- 4 can induce oligodendroglial lineage cells and promote functional recovery in paraplegic mice [13], [16]. Recently, it was demonstrated that transected axons can be regenerated with the adeno-associated virus (AAV) vector encoding BMP- 4 treatment following SCI [81]. In parallel with the available literature reporting the BMP- 4 function in regulating the astrocytes formation, our CAST results showed that the number of BMP- 4^+^ cells were lessened in astrocytes and oligodendrocytes after SCI in the Agm treated group compared with the EC group, suggesting that Agm treatment could be beneficial in controlling glial scar formation through modulating BMPs expression following SCI. Inhibitory role of BMP- 4 in neural differentiation of murine embryonic stem cells further confirmed our immunostaining results of the non-colocalization of BMP- 4^+^ and MAP-2^+^ co-localized cells [82], [83]. While contemplating the BMP- 2/4/7 expression in oligodendrocyte precursor cell population (NG2), our immunofluroscence staining showed no co-localized cells at the injury site (data not shown). However, co-localized cells were identified little away from the injury site and staining pictures suggested that Agm treatment activated NG2^+^/Olig-2^+^ cells and this increase is thought to be associated with NG2^+^/BMP- 2^+^/7^+^cells. The attenuation of NG2^+^/GFAP^+^ cells seems to be related with the decreased population of NG2^+^/BMP- 4^+^ cells.

Immunohistochemical techniques that enable the detection of specific molecular markers at the single-cell level are essential tools for identifying and characterizing cells in healthy and pathological tissue. Previous report demonstrated the random quantification of oligodendrocytes, ependymal and astrocytes in SCI mice [39]. We for the first time provided the un-biased distribution data of MAP-2 (neurons), Olig-2 (oligodendrocytes), GFAP (astrocytes) immunopositive cells and BMP- 2/4/7 expressions in neurons, astrocytes and oligodendrocytes in Th 8–Th 10 segments of the spinal cord by CAST analysis program. Our results suggested that the total number of surviving neurons and oligodendrocytes were increased and the astrocytes poulation was decreased and the total cell number of the surviving cells almost reached almost to that of normal control group in the Agm treated mice at 35DPI. And also, Agm treatment increased the BMP- 2/7 expressions in neurons and oligodendrocytes and decreased in the astrocytes following SCI. Moreover, the reduction of gliosis and glial scar formation following SCI is thought to be controlled with the decreased expression of BMP- 4 in astrocytes in the Agm treated group following SCI.

### Conclusions

Taken together our results suggest that Agm treatment apparently improved the neurological & functional outcomes, improved the remyelination, rescued the damaged neurons and attenuated the glial scar formation after SCI. These beneficial effects are thought to be related with differential expression of BMP- 2/4/7 in neurons, oligodendrocytes and astrocytes with Agm treatment. However, further studies are necessary to elucidate the pharmacokinetics of Agm in the CNS, which may in turn open a new window into the clinical treatment of human SCI.

## Supporting Information

Figure S1 **Agmatine treatment attenuated apoptosis following SCI.** The EC (*n* = 4) and Agm treated group (*n* = 4) were immunostained with p53 antibody at (A) 14 and (B) 35 DPI. The p53 expression was substantially decreased after SCI in the Agm treated group compared with the EC group at 14 and 35 DPI. Scale bars: 50 µm.
(TIF)

Figure S2 **Agmatine treatment increased serotogenic fiber following SCI.** Images were taken from the mice which received either Agm or saline (*n* = 3, per group) following SCI. Agm treated mice showed dense network of 5-HT^+^ serotonergic fibers in the caudal region of the spinal cord almost showing the same morphology to that of the normal control group (*n = *3). EC group showed the beaded and broken morphology of serotogenic fibers both at 14 and 35 DPI. Scale bars: 50 µm.
(TIF)

Figure S3 **Agmatine treatment increased the expansion of oligodendrocyte progenitor cells (NG2^+^) following SCI.** Immunolocalization of NG2^+^ cells in astrocytes (GFAP^+^) and oligodendrocytes (Olig-2^+^) at (A) 7 and (B) 35 DPI. The number of NG2^+^/GFAP^+^ cells were reduced in the Agm treated group (*n* = 5) compared with the EC group (*n* = 5) at (C) 7 & (D) 35 DPI. The NG2^+^/Olig-2^+^ cells expansion were outnumbered in the Agm treated group both at 7 & 35 DPI compared with EC group.
(TIF)

Figure S4 **Agmatine treatment increased the number of oligodendroyctes following SCI.** The quantitative measurements of the total Olig-2^+^ cells by CAST analysis in (A) the rostral (Th 8), (B) lesion (Th 9) and (C) caudal (Th 10) regions after SCI. The results showed a significant increase of the Olig-2^+^ cells in Th 8, Th 9 and Th 10 segments of the injured spinal cord in the Agm treated group compared with the EC group and the values reached significance at 35 DPI (*n* = 5). †, *p*<0.05 NC group vs EC group; #, *p*<0.05 NC group vs Agm treated group; *, *p*<0.05 EC group vs Agm treated group. Results represent mean ± S.E.M.
(TIF)

Figure S5 **Agmatine treatment prevented neuronal cells death following SCI.** The quantitative measurement of the total MAP-2^+^ cells using CAST analysis in (A) the rostral (Th 8), (B) the lesion (Th 9) and (C) the caudal (Th 10) regions of the injured spinal cord (*n* = 5, per group).The results showed an increase of MAP-2^+^ cells in Th 8, Th 9, and Th 10 segments of the spinal cord in the Agm treated group (*n* = 5) compared with the EC group (*n* = 5) and significant increase was recorded at 14 and 35 DPI in rostral and caudal segments. †, *p*<0.05 NC group vs EC group; #, *p*<0.05 NC group vs Agm treated group; *, *p*<0.05 EC group vs Agm treated group. Results represent mean ± S.E.M.
(TIF)

Figure S6 **Agmatine treatment reduced the number of astrocytes following SCI.** The quantitative measurement of the GFAP^+^ cells using CAST analysis in the (A) rostral (Th 8), (B) lesion (Th 9) and (C) caudal (Th 10) regions of injured spinal cord. The results showed a significant decrease of the GFAP^+^ cells in Th 8, Th 9, and Th 10 segments of the injured spinal cord in the Agm treated group (*n* = 5) compared with the EC group (*n* = 5) at 7, 14, and 35 DPI. †, *p*<0.05 NC group vs EC group; #, *p*<0.05 NC group vs Agm treated group; *, *p*<0.05 EC group vs Agm treated group. Results represent mean ± S.E.M.
(TIF)

Figure S7 **Non co-localization of neurons and BMP- 4 following SCI.** There were no BMP- 4 & MAP-2 co-localized cells both in the EC group (*n* = 4) and Agm treated group (*n* = 4) at (A) 7 days and (B) 35 days around the lesion site following SCI. Scale bars: in A, 100 µm & in B, 10 µm.
(TIF)

Figure S8 **Agmatine treatment increased oligodendrocyte progenitor cells (NG2^+^) following SCI.** The expression of NG2^+^ in BMP- 2/4/7^+^ cell population was determined by immunofluorescence staining. The NG2^+^/BMP- 2^+^ cells were higher in the Agm treated group compared with EC group at (A) 7 and (B) 35 DPI. (C) Conversely the expansion of NG2^+^/BMP- 7^+^ cells were increased around the lesion site in Agm treated group at 35 DPI compared to EC group. (D) NG2^+^/BMP- 4^+^ cell population was decreased at 7 days and the (E) NG2^+^/BMP- 4^+^ cells were almost disappeared in the Agm treated group at 35 DPI.
(TIF)

Table S1 **The table showing the allocation of animals in normal control (NC), saline treated mice (EC group) and Agm treated mice (Agm treated group) following SCI.**
(DOCX)

Table S2 **The average numbers of BMP- 2/4/7^+^ cells in neurons and glial cells in Th 8–Th 10 segments following SCI.** The table showing the total number of BMP- 2/4/7^+^ cells co-localized with MAP-2^+^,Olig-2^+^ and GFAP^+^ cell population in Th 8–Th 10 segments at 1, 7, 14, and 35 DPI in NC group (*n* = 5), EC group (*n* = 5) and Agm treated group (*n* = 5) using computer assisted stereological toolbox (CAST) analysis. The immunopositive cells were measured per mm^3^ (Units:×10^6^cells/mm^3^). †, *p*<0.05 NC group vs EC group; #, *p*<0.05 NC group vs Agm treated group; *, *p*<0.05 EC group vs Agm treated group. Results represent mean ± S.E.M.
(DOCX)

## References

[1] Guertin PA (2008) Anxiolytics may promote locomotor function recovery in spinal cord injury patients. Neuropsychiatr Dis Treat 4: 759–763. 19043520 10.2147/ndt.s2839 PMC2536543

[2] Yoshimura N (1999) Bladder afferent pathway and spinal cord injury: possible mechanisms inducing hyperreflexia of the urinary bladder. Prog Neurobiol 57: 583–606. 10221783 10.1016/s0301-0082(98)00070-7

[3] Rasouli A, Bhatia N, Dinh P, Cahill K, Suryadevara S, et al (2009) Resection of glial scar following spinal cord injury. J Orthop Res 27: 931–936. 19062171 10.1002/jor.20793 PMC2696557

[4] McDonald JW, Belegu V (2006) Demyelination and remyelination after spinal cord injury. J Neurotrauma 23: 345–359. 16629621 10.1089/neu.2006.23.345

[5] Kotter MR, Stadelmann C, Hartung HP (2011) Enhancing remyelination in disease–can we wrap it up? Brain 134: 1882–1900. 21507994 10.1093/brain/awr014

[6] Kulbatski I, Mothe AJ, Keating A, Hakamata Y, Kobayashi E, et al (2007) Oligodendrocytes and radial glia derived from adult rat spinal cord progenitors: morphological and immunocytochemical characterization. J Histochem Cytochem 55: 209–222. 17101728 10.1369/jhc.6A7020.2006

[7] Li G, Regunathan S, Barrow CJ, Eshraghi J, Cooper R, et al (1994) Agmatine: an endogenous clonidine-displacing substance in the brain. Science 263: 966–969. 7906055 10.1126/science.7906055

[8] Piletz JE, Chikkala DN, Ernsberger P (1995) Comparison of the properties of agmatine and endogenous clonidine-displacing substance at imidazoline and alpha-2 adrenergic receptors. J Pharmacol Exp Ther 272: 581–587. 7853171

[9] Murayama T, Tsai SC, Adamik R, Moss J, Vaughan M (1993) Effects of temperature on ADP-ribosylation factor stimulation of cholera toxin activity. Biochemistry 32: 561–566. 8422366 10.1021/bi00053a022

[10] Fairbanks CA, Schreiber KL, Brewer KL, Yu CG, Stone LS, et al (2000) Agmatine reverses pain induced by inflammation, neuropathy, and spinal cord injury. Proc Natl Acad Sci U S A 97: 10584–10589. 10984543 10.1073/pnas.97.19.10584 PMC27068

[11] Chen D, Zhao M, Mundy GR (2004) Bone morphogenetic proteins. Growth Factors 22: 233–241. 15621726 10.1080/08977190412331279890

[12] Karimi-Abdolrezaee S, Schut D, Wang J, Fehlings MG (2012) Chondroitinase and growth factors enhance activation and oligodendrocyte differentiation of endogenous neural precursor cells after spinal cord injury. PLoS One 7: e37589. 22629425 10.1371/journal.pone.0037589 PMC3358255

[13] Obermair FJ, Schroter A, Thallmair M (2008) Endogenous neural progenitor cells as therapeutic target after spinal cord injury. Physiology (Bethesda) 23: 296–304. 18927205 10.1152/physiol.00017.2008

[14] Reiriz J, Espejo M, Ventura F, Ambrosio S, Alberch J (1999) Bone morphogenetic protein-2 promotes dissociated effects on the number and differentiation of cultured ventral mesencephalic dopaminergic neurons. J Neurobiol 38: 161–170. 10022564

[15] Lein PJ, Beck HN, Chandrasekaran V, Gallagher PJ, Chen HL, et al (2002) Glia induce dendritic growth in cultured sympathetic neurons by modulating the balance between bone morphogenetic proteins (BMPs) and BMP antagonists. J Neurosci 22: 10377–10387. 12451137 10.1523/JNEUROSCI.22-23-10377.2002 PMC6758753

[16] Xiao Q, Du Y, Wu W, Yip HK (2010) Bone morphogenetic proteins mediate cellular response and, together with Noggin, regulate astrocyte differentiation after spinal cord injury. Exp Neurol 221: 353–366. 20005873 10.1016/j.expneurol.2009.12.003

[17] de Rivero Vaccari JP, Marcillo A, Nonner D, Dietrich WD, Keane RW (2009) Neuroprotective effects of bone morphogenetic protein 7 (BMP7) treatment after spinal cord injury. Neurosci Lett 465: 226–229. 19765637 10.1016/j.neulet.2009.09.013

[18] Setoguchi T, Yone K, Matsuoka E, Takenouchi H, Nakashima K, et al (2001) Traumatic injury-induced BMP7 expression in the adult rat spinal cord. Brain Res 921: 219–225. 11720729 10.1016/s0006-8993(01)03123-7

[19] Chalazonitis A, D'Autreaux F, Pham TD, Kessler JA, Gershon MD (2011) Bone morphogenetic proteins regulate enteric gliogenesis by modulating ErbB3 signaling. Dev Biol 350: 64–79. 21094638 10.1016/j.ydbio.2010.11.017 PMC3034360

[20] See J, Mamontov P, Ahn K, Wine-Lee L, Crenshaw EB III, et al (2007) BMP signaling mutant mice exhibit glial cell maturation defects. Mol Cell Neurosci 35: 171–182. 17391983 10.1016/j.mcn.2007.02.012 PMC1950488

[21] Matsuura I, Taniguchi J, Hata K, Saeki N, Yamashita T (2008) BMP inhibition enhances axonal growth and functional recovery after spinal cord injury. J Neurochem 105: 1471–1479. 18221366 10.1111/j.1471-4159.2008.05251.x

[22] Setoguchi T, Nakashima K, Takizawa T, Yanagisawa M, Ochiai W, et al (2004) Treatment of spinal cord injury by transplantation of fetal neural precursor cells engineered to express BMP inhibitor. Exp Neurol 189: 33–44. 15296834 10.1016/j.expneurol.2003.12.007

[23] Kim JH, Lee YW, Park YM, Park KA, Park SH, et al (2011) Agmatine-reduced collagen scar area accompanied with surface righting reflex recovery after complete transection spinal cord injury. Spine (Phila Pa 1976) 36: 2130–2138. 21325984 10.1097/BRS.0b013e318205e3f7

[24] Basso DM, Fisher LC, Anderson AJ, Jakeman LB, McTigue DM, et al (2006) Basso Mouse Scale for locomotion detects differences in recovery after spinal cord injury in five common mouse strains. J Neurotrauma 23: 635–659. 16689667 10.1089/neu.2006.23.635

[25] Pajoohesh-Ganji A, Byrnes KR, Fatemi G, Faden AI (2010) A combined scoring method to assess behavioral recovery after mouse spinal cord injury. Neurosci Res 67: 117–125. 20188770 10.1016/j.neures.2010.02.009 PMC2879004

[26] Sharp KG, Dickson AR, Marchenko SA, Yee KM, Emery PN, et al (2012) Salmon fibrin treatment of spinal cord injury promotes functional recovery and density of serotonergic innervation. Exp Neurol 235: 345–356. 22414309 10.1016/j.expneurol.2012.02.016 PMC3437931

[27] Hvid M, Jensen HK, Deleuran B, Kemp K, Andersson C, et al (2009) Evaluation of FITC-induced atopic dermatitis-like disease in NC/Nga mice and BALB/c mice using computer-assisted stereological toolbox, a computer-aided morphometric system. Int Arch Allergy Immunol 149: 188–194. 19218811 10.1159/000199714

[28] de Groat WC, Yoshimura N (2006) Mechanisms underlying the recovery of lower urinary tract function following spinal cord injury. Prog Brain Res 152: 59–84. 16198694 10.1016/S0079-6123(05)52005-3

[29] Widerstrom-Noga E, Cruz-Almeida Y, Krassioukov A (2004) Is there a relationship between chronic pain and autonomic dysreflexia in persons with cervical spinal cord injury? J Neurotrauma 21: 195–204. 15000760 10.1089/089771504322778659

[30] Estores IM (2003) The consumer's perspective and the professional literature: what do persons with spinal cord injury want? J Rehabil Res Dev 40: 93–98. 15077653 10.1682/jrrd.2003.08.0093

[31] Anderson KD (2004) Targeting recovery: priorities of the spinal cord-injured population. J Neurotrauma 21: 1371–1383. 15672628 10.1089/neu.2004.21.1371

[32] Amar AP, Levy ML (1999) Pathogenesis and pharmacological strategies for mitigating secondary damage in acute spinal cord injury. Neurosurgery 44: 1027–1039. 10232536 10.1097/00006123-199905000-00052

[33] Crowe MJ, Bresnahan JC, Shuman SL, Masters JN, Beattie MS (1997) Apoptosis and delayed degeneration after spinal cord injury in rats and monkeys. Nat Med 3: 73–76. 8986744 10.1038/nm0197-73

[34] Thuret S, Moon LD, Gage FH (2006) Therapeutic interventions after spinal cord injury. Nat Rev Neurosci 7: 628–643. 16858391 10.1038/nrn1955

[35] Bregman BS, Kunkel-Bagden E, Reier PJ, Dai HN, McAtee M, et al (1993) Recovery of function after spinal cord injury: mechanisms underlying transplant-mediated recovery of function differ after spinal cord injury in newborn and adult rats. Exp Neurol 123: 3–16. 8405277 10.1006/exnr.1993.1136

[36] Nygren LG, Fuxe K, Jonsson G, Olson L (1974) Functional regeneration of 5-hydroxytryptamine nerve terminals in the rat spinal cord following 5, 6-dihydroxytryptamine induced degeneration. Brain Res 78: 377–394. 4424711 10.1016/0006-8993(74)90922-6

[37] Shoichet MS (2008) Strategies for Regeneration and Repair in the Injured Central Neurous System. Taylor & Francis Group, LLC.CRC press. 221 p. 21204406

[38] Boido M, Garbossa D, Vercelli A (2011) Early graft of neural precursors in spinal cord compression reduces glial cyst and improves function. J Neurosurg Spine 15: 97–106. 21456892 10.3171/2011.1.SPINE10607

[39] Barnabe-Heider F, Goritz C, Sabelstrom H, Takebayashi H, Pfrieger FW, et al (2010) Origin of new glial cells in intact and injured adult spinal cord. Cell Stem Cell 7: 470–482. 20887953 10.1016/j.stem.2010.07.014

[40] Choi BH (1986) Myelin-forming oligodendrocytes of developing mouse spinal cord: immunocytochemical and ultrastructural studies. J Neuropathol Exp Neurol 45: 513–524. 2427660 10.1097/00005072-198609000-00003

[41] Wu J, Yoo S, Wilcock D, Lytle JM, Leung PY, et al (2010) Interaction of NG2(+) glial progenitors and microglia/macrophages from the injured spinal cord. Glia 58: 410–422. 19780197 10.1002/glia.20932 PMC2807472

[42] Lytle JM, Wrathall JR (2007) Glial cell loss, proliferation and replacement in the contused murine spinal cord. Eur J Neurosci 25: 1711–1724. 17432960 10.1111/j.1460-9568.2007.05390.x

[43] Ille F, Atanasoski S, Falk S, Ittner LM, Marki D, et al (2007) Wnt/BMP signal integration regulates the balance between proliferation and differentiation of neuroepithelial cells in the dorsal spinal cord. Dev Biol 304: 394–408. 17292876 10.1016/j.ydbio.2006.12.045

[44] Fuller ML, DeChant AK, Rothstein B, Caprariello A, Wang R, et al (2007) Bone morphogenetic proteins promote gliosis in demyelinating spinal cord lesions. Ann Neurol 62: 288–300. 17696121 10.1002/ana.21179

[45] Gomes WA, Mehler MF, Kessler JA (2003) Transgenic overexpression of BMP4 increases astroglial and decreases oligodendroglial lineage commitment. Dev Biol 255: 164–177. 12618141 10.1016/s0012-1606(02)00037-4

[46] Auguet M, Viossat I, Marin JG, Chabrier PE (1995) Selective inhibition of inducible nitric oxide synthase by agmatine. Jpn J Pharmacol 69: 285–287. 8699639 10.1254/jjp.69.285

[47] Galea E, Regunathan S, Eliopoulos V, Feinstein DL, Reis DJ (1996) Inhibition of mammalian nitric oxide synthases by agmatine, an endogenous polyamine formed by decarboxylation of arginine. Biochem J 316 (Pt 1): 247–249. 10.1042/bj3160247 PMC1217329 8645212

[48] Halaris A, Plietz J (2007) Agmatine : metabolic pathway and spectrum of activity in brain. CNS Drugs 21: 885–900. 17927294 10.2165/00023210-200721110-00002

[49] Yu CG, Marcillo AE, Fairbanks CA, Wilcox GL, Yezierski RP (2000) Agmatine improves locomotor function and reduces tissue damage following spinal cord injury. Neuroreport 11: 3203–3207. 11043549 10.1097/00001756-200009280-00031

[50] Shunmugavel A (2010) Simvastatin protects bladder and renal functions following spinal cord injury in rats. J Inflamm (Lond). 10.1186/1476-9255-7-17 PMC2873501 20403180

[51] Silver J, Miller JH (2004) Regeneration beyond the glial scar. Nat Rev Neurosci 5: 146–156. 14735117 10.1038/nrn1326

[52] Franklin RJ, Ffrench-Constant C (2008) Remyelination in the CNS: from biology to therapy. Nat Rev Neurosci 9: 839–855. 18931697 10.1038/nrn2480

[53] Jarjour AA, Zhang H, Bauer N, Ffrench-Constant C, Williams A (2012) In vitro modeling of central nervous system myelination and remyelination. Glia 60: 1–12. 21858876 10.1002/glia.21231

[54] Wu B, Ren X (2009) Promoting axonal myelination for improving neurological recovery in spinal cord injury. J Neurotrauma 26: 1847–1856. 19785544 10.1089/neu.2008.0551

[55] Keirstead HS, Blakemore WF (1997) Identification of post-mitotic oligodendrocytes incapable of remyelination within the demyelinated adult spinal cord. J Neuropathol Exp Neurol 56: 1191–1201. 9370229 10.1097/00005072-199711000-00003

[56] Redwine JM, Armstrong RC (1998) In vivo proliferation of oligodendrocyte progenitors expressing PDGFalphaR during early remyelination. J Neurobiol 37: 413–428. 9828047 10.1002/(sici)1097-4695(19981115)37:3<413::aid-neu7>3.0.co;2-8

[57] Chang A, Nishiyama A, Peterson J, Prineas J, Trapp BD (2000) NG2-positive oligodendrocyte progenitor cells in adult human brain and multiple sclerosis lesions. J Neurosci 20: 6404–6412. 10964946 10.1523/JNEUROSCI.20-17-06404.2000 PMC6772992

[58] Nishiyama A (2007) Polydendrocytes: NG2 cells with many roles in development and repair of the CNS. Neuroscientist 13: 62–76. 17229976 10.1177/1073858406295586

[59] Cigognini D, Satta A, Colleoni B, Silva D, Donega M, et al (2011) Evaluation of early and late effects into the acute spinal cord injury of an injectable functionalized self-assembling scaffold. PLoS One 6: e19782. 21611127 10.1371/journal.pone.0019782 PMC3097206

[60] Butt SJ, Harris-Warrick RM, Kiehn O (2002) Firing properties of identified interneuron populations in the mammalian hindlimb central pattern generator. J Neurosci 22: 9961–9971. 12427853 10.1523/JNEUROSCI.22-22-09961.2002 PMC6757818

[61] Grillner S, Zangger P (1979) On the central generation of locomotion in the low spinal cat. Exp Brain Res 34: 241–261. 421750 10.1007/BF00235671

[62] Jacobs BL, Martin-Cora FJ, Fornal CA (2002) Activity of medullary serotonergic neurons in freely moving animals. Brain Res Brain Res Rev 40: 45–52. 12589905 10.1016/s0165-0173(02)00187-x

[63] Jordan LM, Liu J, Hedlund PB, Akay T, Pearson KG (2008) Descending command systems for the initiation of locomotion in mammals. Brain Res Rev 57: 183–191. 17928060 10.1016/j.brainresrev.2007.07.019

[64] Fouad K, Rank MM, Vavrek R, Murray KC, Sanelli L, et al (2010) Locomotion after spinal cord injury depends on constitutive activity in serotonin receptors. J Neurophysiol 104: 2975–2984. 20861436 10.1152/jn.00499.2010 PMC3007654

[65] Saruhashi Y, Matsusue Y, Fujimiya M (2009) The recovery of 5-HT transporter and 5-HT immunoreactivity in injured rat spinal cord. Arch Orthop Trauma Surg 129: 1279–1285. 18825396 10.1007/s00402-008-0754-z

[66] Boulenguez P, Vinay L (2009) Strategies to restore motor functions after spinal cord injury. Curr Opin Neurobiol 19: 587–600. 19896827 10.1016/j.conb.2009.10.005

[67] Hawryluk GW, Rowland J, Kwon BK, Fehlings MG (2008) Protection and repair of the injured spinal cord: a review of completed, ongoing, and planned clinical trials for acute spinal cord injury. Neurosurg Focus 25: E14. 10.3171/FOC.2008.25.11.E14 18980474

[68] do Carmo Cunha J, de Freitas Azevedo Levy B, de Luca BA, de Andrade MS, Gomide VC, et al (2007) Responses of reactive astrocytes containing S100beta protein and fibroblast growth factor-2 in the border and in the adjacent preserved tissue after a contusion injury of the spinal cord in rats: implications for wound repair and neuroregeneration. Wound Repair Regen 15: 134–146. 17244329 10.1111/j.1524-475X.2006.00194.x

[69] Basso DM, Beattie MS, Bresnahan JC (1996) Graded histological and locomotor outcomes after spinal cord contusion using the NYU weight-drop device versus transection. Exp Neurol 139: 244–256. 8654527 10.1006/exnr.1996.0098

[70] Hampton DW, Asher RA, Kondo T, Steeves JD, Ramer MS, et al (2007) A potential role for bone morphogenetic protein signalling in glial cell fate determination following adult central nervous system injury in vivo. Eur J Neurosci 26: 3024–3035. 18028109 10.1111/j.1460-9568.2007.05940.x

[71] Tsujii M, Akeda K, Iino T, Uchida A (2009) Are BMPs involved in normal nerve and following transection?: a pilot study. Clin Orthop Relat Res 467: 3183–3189. 19669850 10.1007/s11999-009-1009-1 PMC2772907

[72] Davies JE, Proschel C, Zhang N, Noble M, Mayer-Proschel M, et al (2008) Transplanted astrocytes derived from BMP- or CNTF-treated glial-restricted precursors have opposite effects on recovery and allodynia after spinal cord injury. J Biol 7: 24. 18803859 10.1186/jbiol85 PMC2776404

[73] Liu A, Niswander LA (2005) Bone morphogenetic protein signalling and vertebrate nervous system development. Nat Rev Neurosci 6: 945–954. 16340955 10.1038/nrn1805

[74] Henriquez JP, Krull CE, Osses N (2011) The Wnt and BMP Families of Signaling Morphogens at the Vertebrate Neuromuscular Junction. Int J Mol Sci 12: 8924–8946. 22272112 10.3390/ijms12128924 PMC3257109

[75] Pisano JM, Colon-Hastings F, Birren SJ (2000) Postmigratory enteric and sympathetic neural precursors share common, developmentally regulated, responses to BMP2. Dev Biol 227: 1–11. 11076672 10.1006/dbio.2000.9876

[76] Nakashima K, Takizawa T, Ochiai W, Yanagisawa M, Hisatsune T, et al (2001) BMP2-mediated alteration in the developmental pathway of fetal mouse brain cells from neurogenesis to astrocytogenesis. Proc Natl Acad Sci U S A 98: 5868–5873. 11331769 10.1073/pnas.101109698 PMC33305

[77] Chang CF, Lin SZ, Chiang YH, Morales M, Chou J, et al (2003) Intravenous administration of bone morphogenetic protein-7 after ischemia improves motor function in stroke rats. Stroke 34: 558–564. 12574575 10.1161/01.str.0000051507.64423.00

[78] Harvey BK, Hoffer BJ, Wang Y (2005) Stroke and TGF-beta proteins: glial cell line-derived neurotrophic factor and bone morphogenetic protein. Pharmacol Ther 105: 113–125. 15670622 10.1016/j.pharmthera.2004.09.003

[79] Reissmann E, Ernsberger U, Francis-West PH, Rueger D, Brickell PM, et al (1996) Involvement of bone morphogenetic protein-4 and bone morphogenetic protein-7 in the differentiation of the adrenergic phenotype in developing sympathetic neurons. Development 122: 2079–2088. 8681789 10.1242/dev.122.7.2079

[80] White RE, Jakeman LB (2008) Don't fence me in: harnessing the beneficial roles of astrocytes for spinal cord repair. Restor Neurol Neurosci 26: 197–214. 18820411 PMC2825119

[81] Parikh P, Hao Y, Hosseinkhani M, Patil SB, Huntley GW, et al (2011) Regeneration of axons in injured spinal cord by activation of bone morphogenetic protein/Smad1 signaling pathway in adult neurons. Proc Natl Acad Sci U S A 108: E99–107. 21518886 10.1073/pnas.1100426108 PMC3093464

[82] Finley MF, Devata S, Huettner JE (1999) BMP-4 inhibits neural differentiation of murine embryonic stem cells. J Neurobiol 40: 271–287. 10440729

[83] Gambaro K, Aberdam E, Virolle T, Aberdam D, Rouleau M (2006) BMP-4 induces a Smad-dependent apoptotic cell death of mouse embryonic stem cell-derived neural precursors. Cell Death Differ 13: 1075–1087. 16311513 10.1038/sj.cdd.4401799

