# Supplementary figures and images for: The Multifaceted Effects of Agmatine on Functional Recovery after Spinal Cord Injury through Modulations of BMP-2/4/7 Expressions in Neurons and Glial Cells
Source: PLoS One. 2013 Jan 21;8(1):e53911. doi: 10.1371/journal.pone.0053911 (PMC3549976; doi:10.1371/journal.pone.0053911)

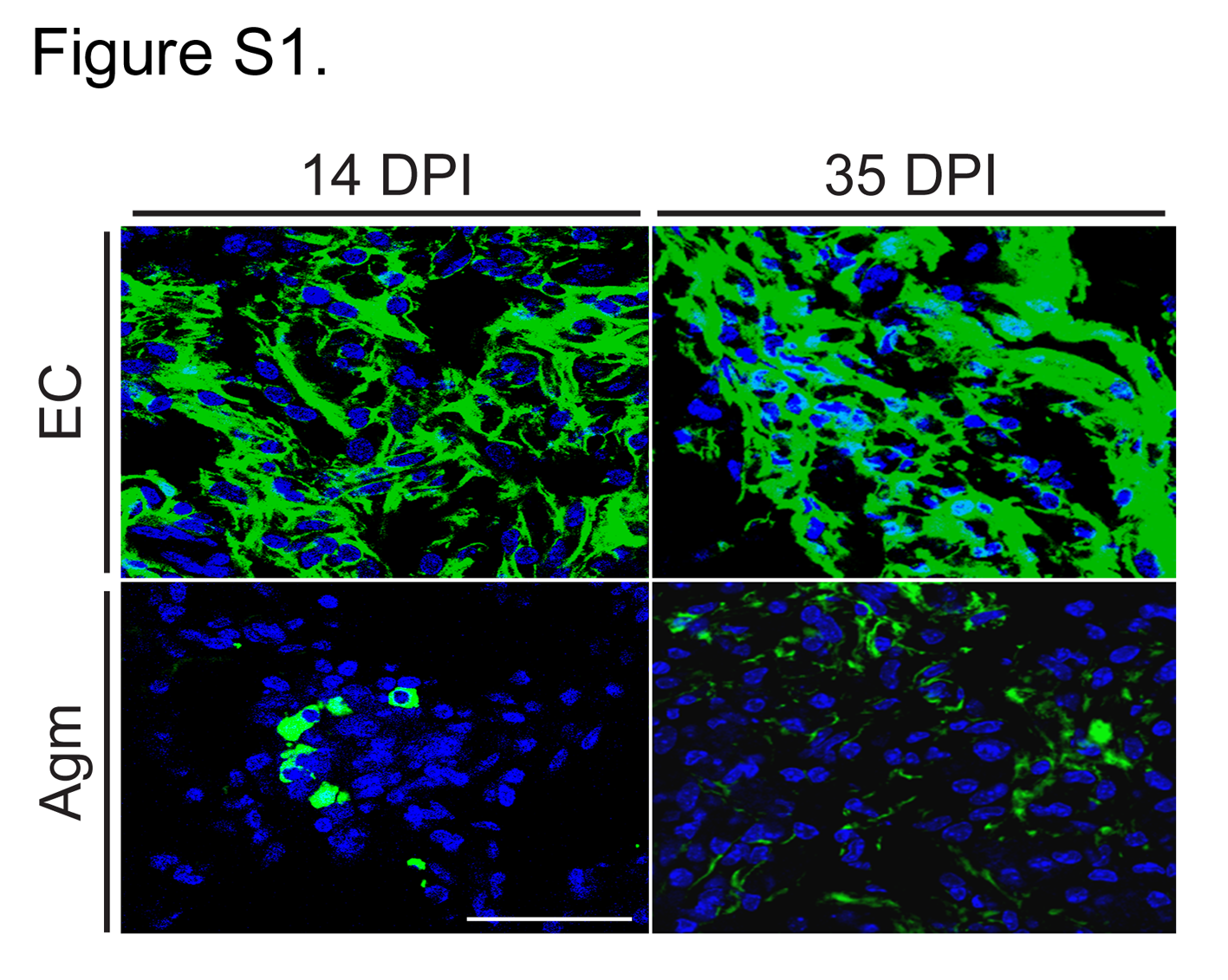

Supplement: Figure S1 — Agmatine treatment attenuated apoptosis following SCI. The EC (n = 4) and Agm treated group (n = 4) were immunostained with p53 antibody at (A) 14 and (B) 35 DPI. The p53 expression was substantially decreased after SCI in the Agm treated group compared with the EC group at 14 and 35 DPI. Scale bars: 50 µm. (TIF) [file pone.0053911.s001.tif]

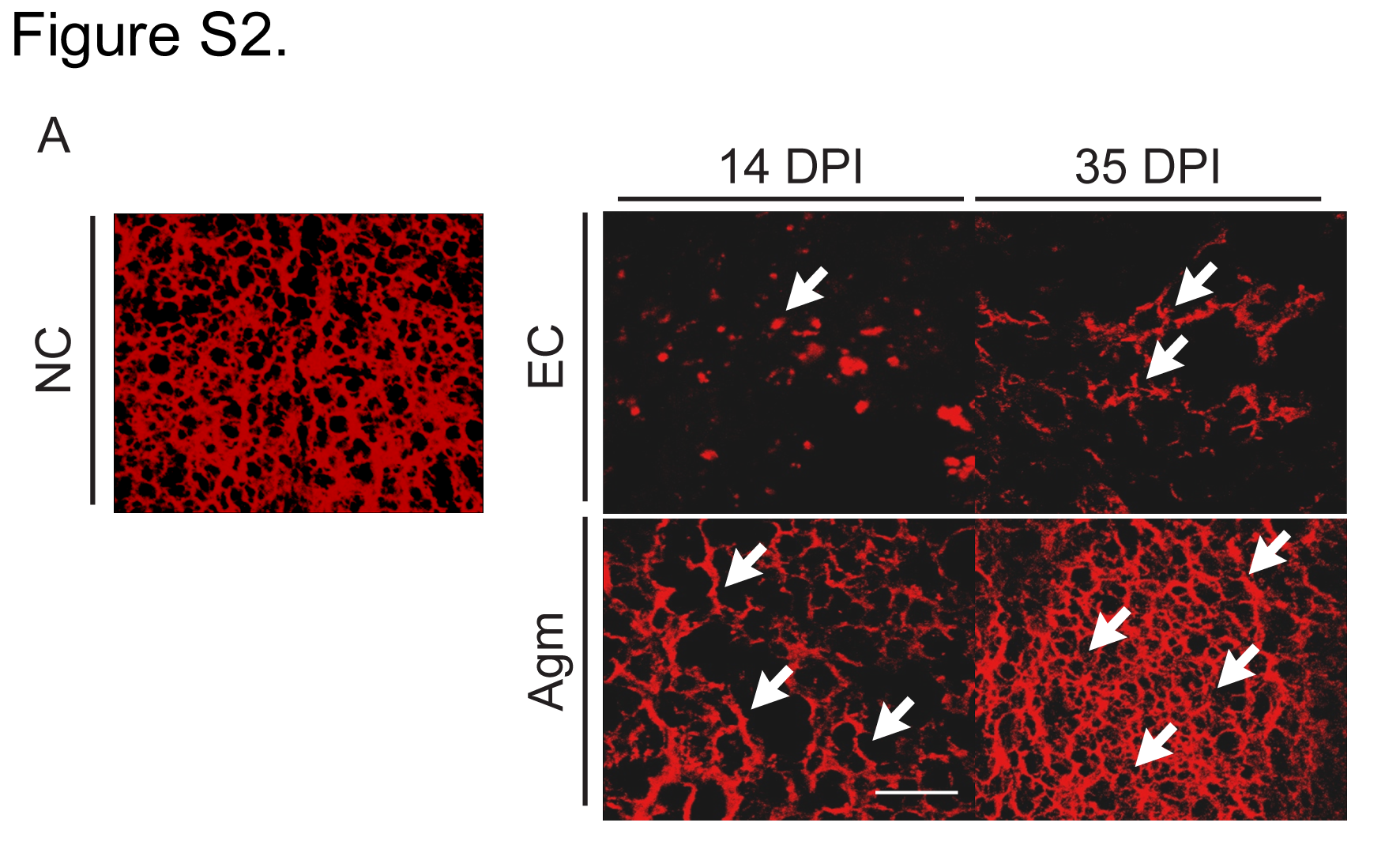

Supplement: Figure S2 — Agmatine treatment increased serotogenic fiber following SCI. Images were taken from the mice which received either Agm or saline (n = 3, per group) following SCI. Agm treated mice showed dense network of 5-HT+ serotonergic fibers in the caudal region of the spinal cord almost showing the same morphology to that of the normal control group (n = 3). EC group showed the beaded and broken morphology of serotogenic fibers both at 14 and 35 DPI. Scale bars: 50 µm. (TIF) [file pone.0053911.s002.tif]

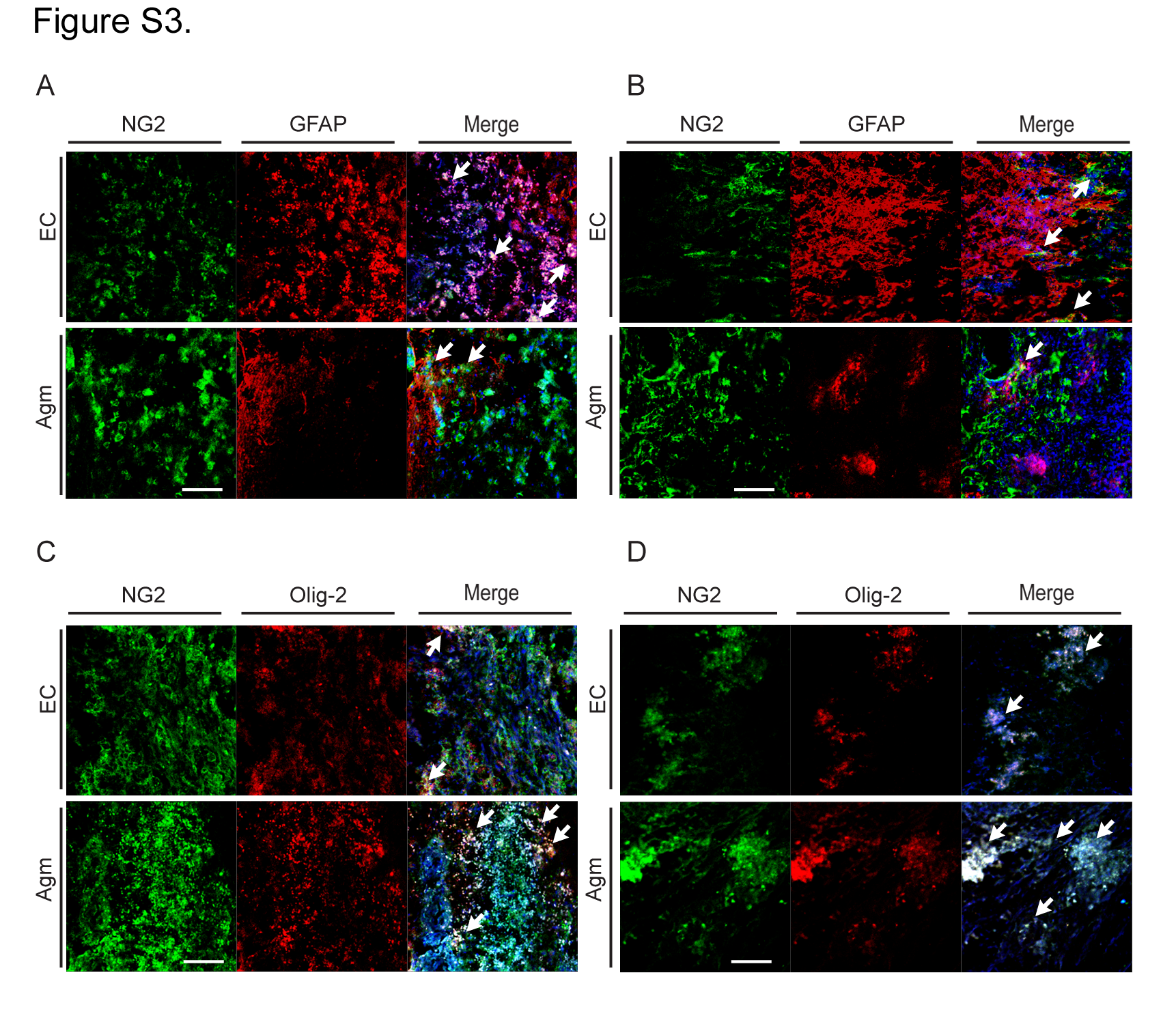

Supplement: Figure S3 — Agmatine treatment increased the expansion of oligodendrocyte progenitor cells (NG2+) following SCI. Immunolocalization of NG2+ cells in astrocytes (GFAP+) and oligodendrocytes (Olig-2+) at (A) 7 and (B) 35 DPI. The number of NG2+/GFAP+ cells were reduced in the Agm treated group (n = 5) compared with the EC group (n = 5) at (C) 7 & (D) 35 DPI. The NG2+/Olig-2+ cells expansion were outnumbered in the Agm treated group both at 7 & 35 DPI compared with EC group. (TIF) [file pone.0053911.s003.tif]

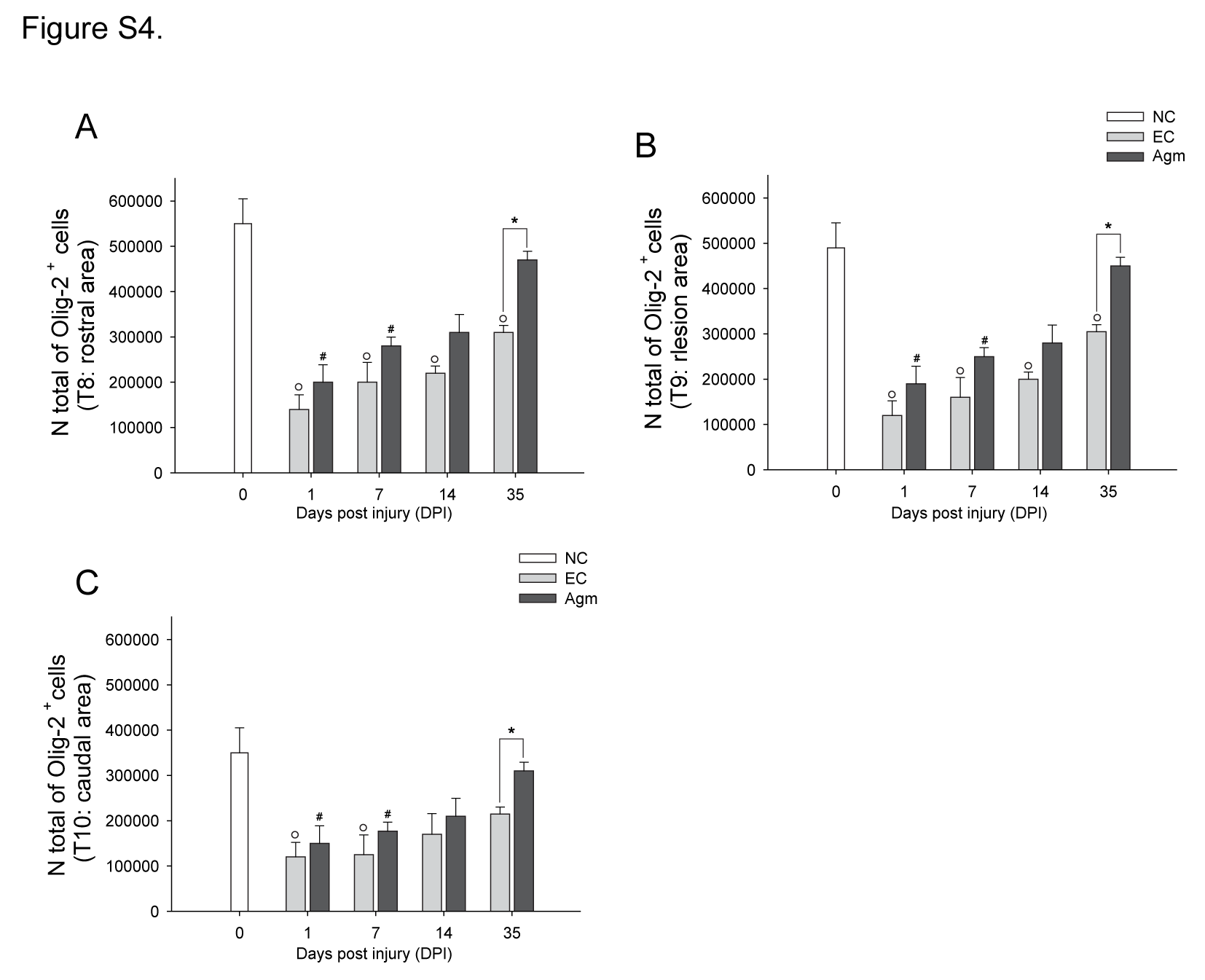

Supplement: Figure S4 — Agmatine treatment increased the number of oligodendroyctes following SCI. The quantitative measurements of the total Olig-2+ cells by CAST analysis in (A) the rostral (Th 8), (B) lesion (Th 9) and (C) caudal (Th 10) regions after SCI. The results showed a significant increase of the Olig-2+ cells in Th 8, Th 9 and Th 10 segments of the injured spinal cord in the Agm treated group compared with the EC group and the values reached significance at 35 DPI (n = 5). †, p<0.05 NC group vs EC group; #, p<0.05 NC group vs Agm treated group; *, p<0.05 EC group vs Agm treated group. Results represent mean ± S.E.M. (TIF) [file pone.0053911.s004.tif]

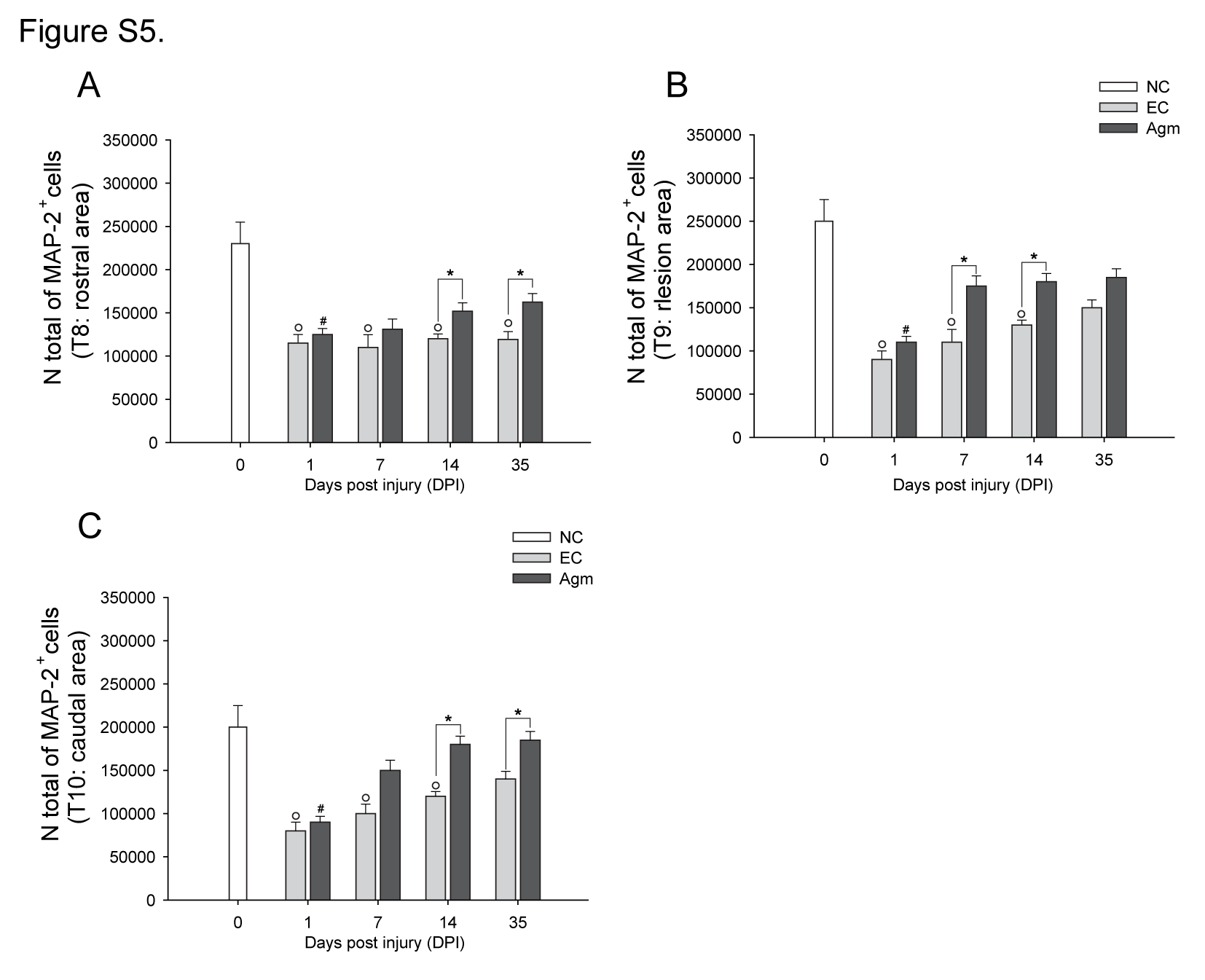

Supplement: Figure S5 — Agmatine treatment prevented neuronal cells death following SCI. The quantitative measurement of the total MAP-2+ cells using CAST analysis in (A) the rostral (Th 8), (B) the lesion (Th 9) and (C) the caudal (Th 10) regions of the injured spinal cord (n = 5, per group).The results showed an increase of MAP-2+ cells in Th 8, Th 9, and Th 10 segments of the spinal cord in the Agm treated group (n = 5) compared with the EC group (n = 5) and significant increase was recorded at 14 and 35 DPI in rostral and caudal segments. †, p<0.05 NC group vs EC group; #, p<0.05 NC group vs Agm treated group; *, p<0.05 EC group vs Agm treated group. Results represent mean ± S.E.M. (TIF) [file pone.0053911.s005.tif]

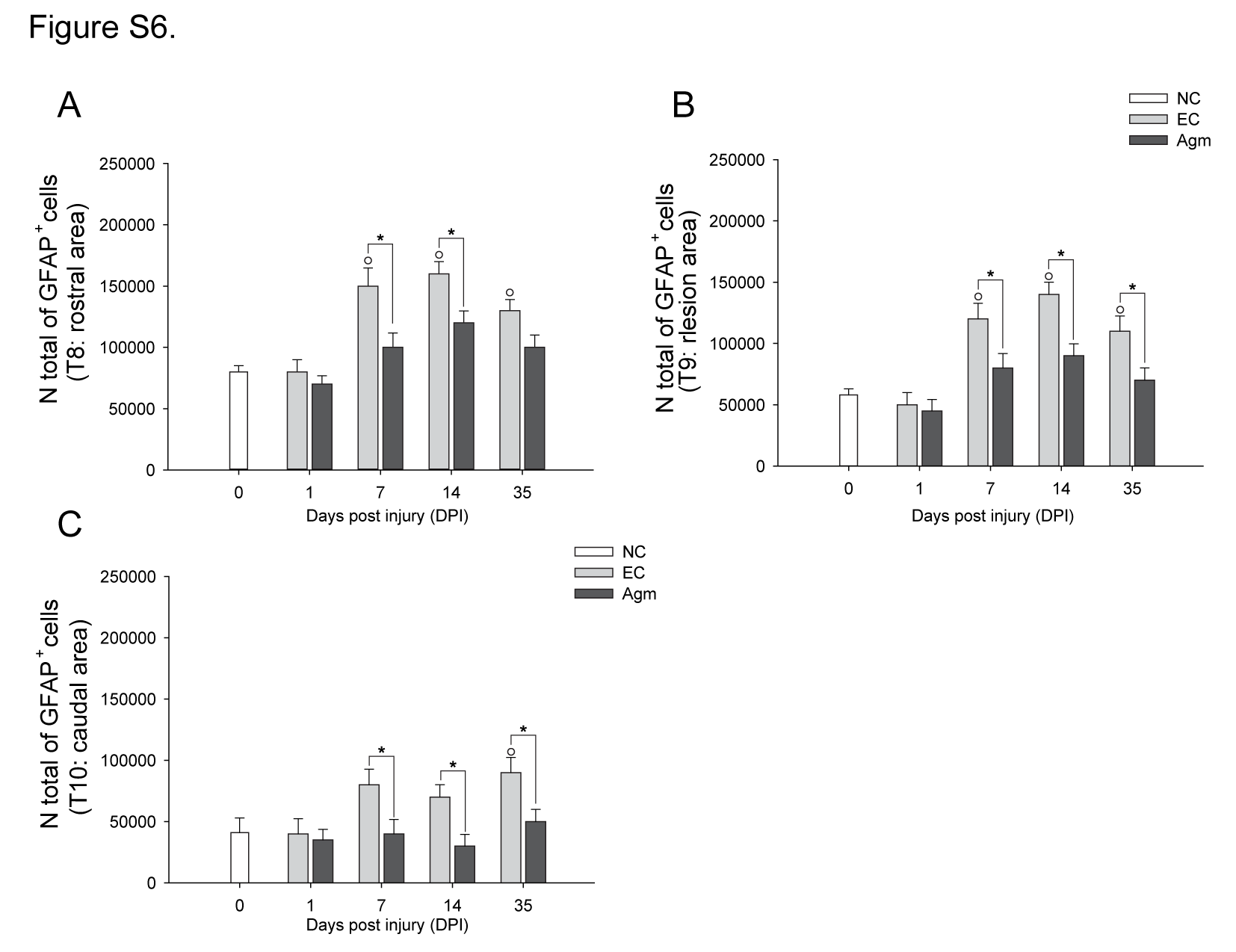

Supplement: Figure S6 — Agmatine treatment reduced the number of astrocytes following SCI. The quantitative measurement of the GFAP+ cells using CAST analysis in the (A) rostral (Th 8), (B) lesion (Th 9) and (C) caudal (Th 10) regions of injured spinal cord. The results showed a significant decrease of the GFAP+ cells in Th 8, Th 9, and Th 10 segments of the injured spinal cord in the Agm treated group (n = 5) compared with the EC group (n = 5) at 7, 14, and 35 DPI. †, p<0.05 NC group vs EC group; #, p<0.05 NC group vs Agm treated group; *, p<0.05 EC group vs Agm treated group. Results represent mean ± S.E.M. (TIF) [file pone.0053911.s006.tif]

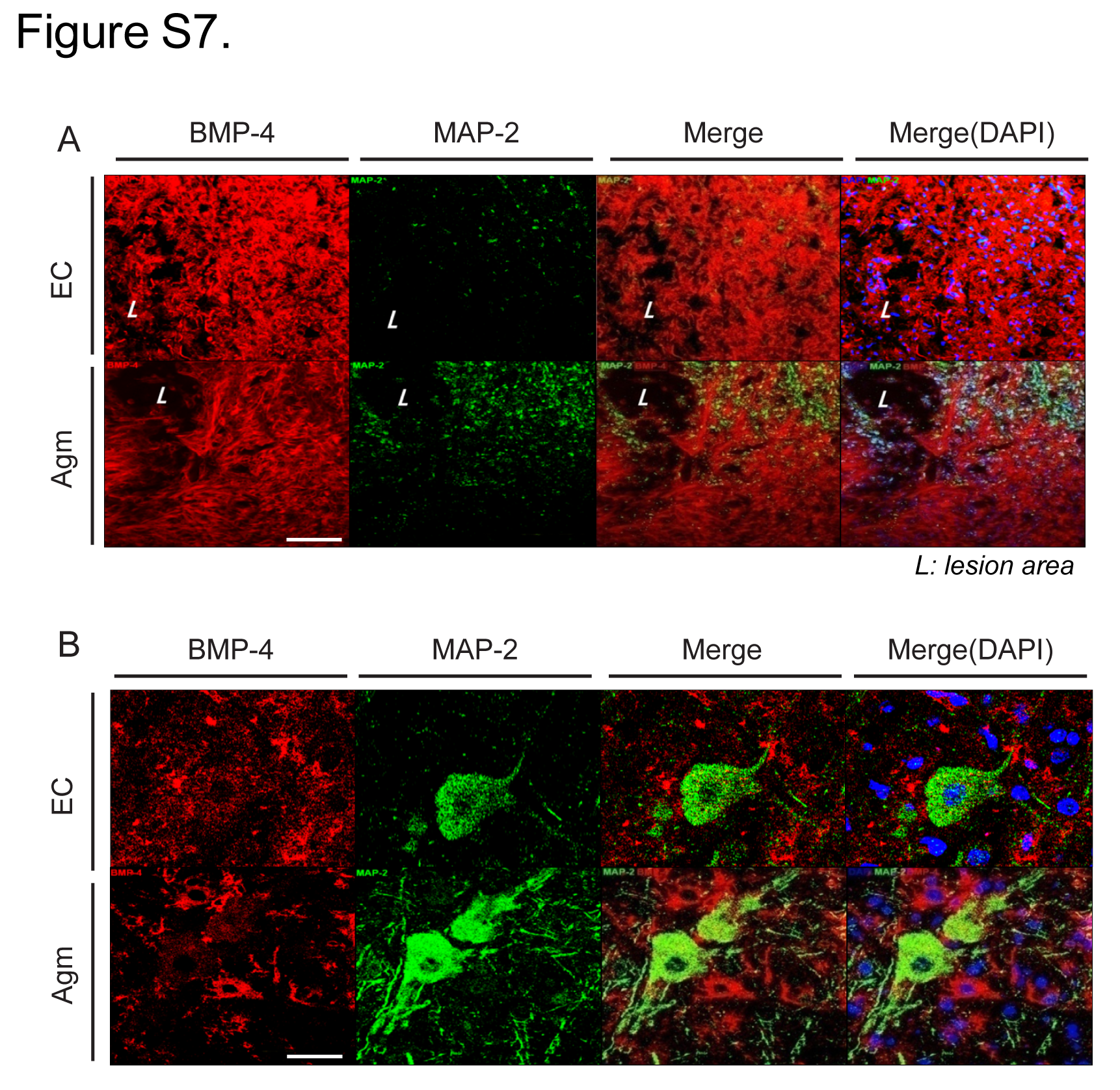

Supplement: Figure S7 — Non co-localization of neurons and BMP- 4 following SCI. There were no BMP- 4 & MAP-2 co-localized cells both in the EC group (n = 4) and Agm treated group (n = 4) at (A) 7 days and (B) 35 days around the lesion site following SCI. Scale bars: in A, 100 µm & in B, 10 µm. (TIF) [file pone.0053911.s007.tif]

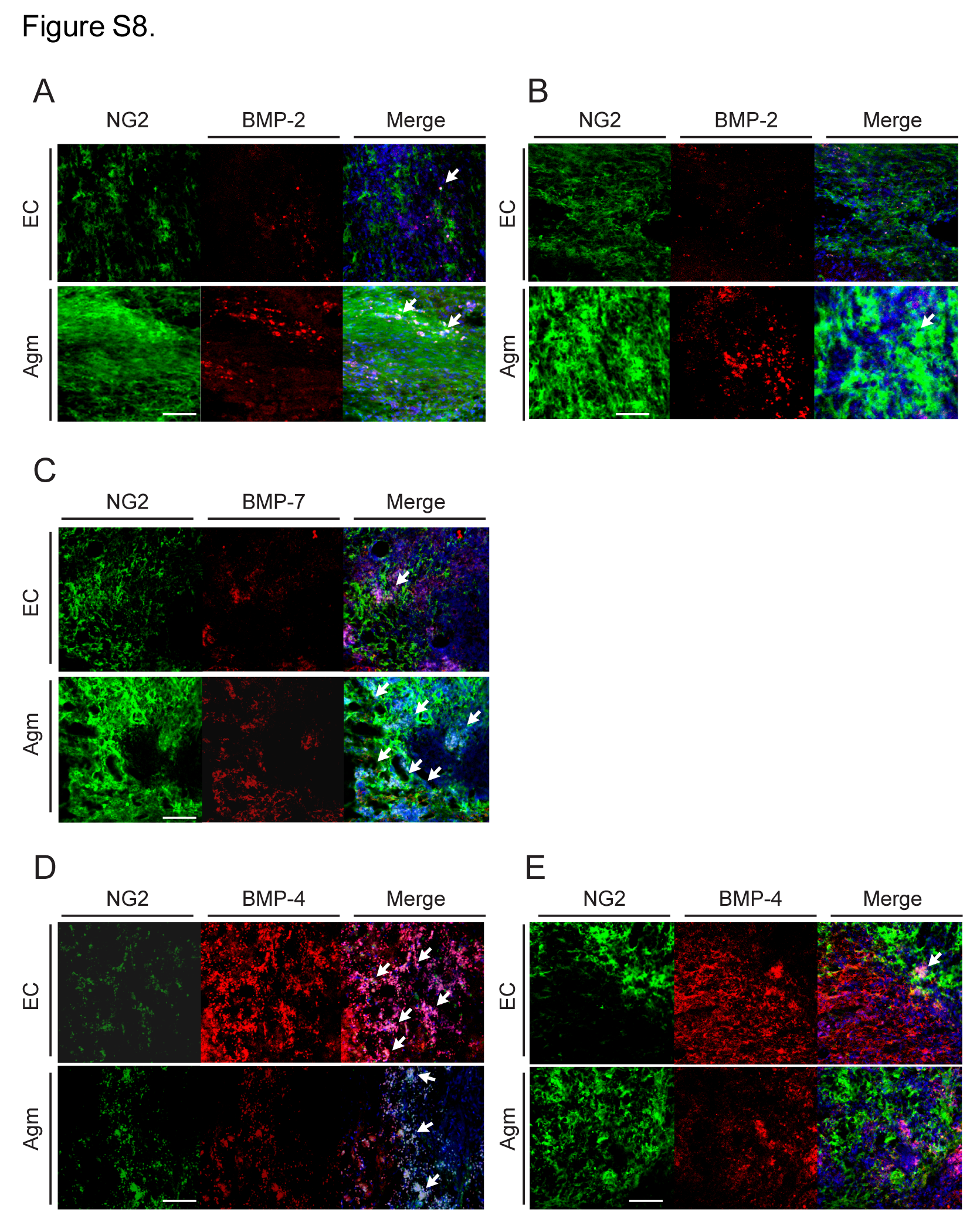

Supplement: Figure S8 — Agmatine treatment increased oligodendrocyte progenitor cells (NG2+) following SCI. The expression of NG2+ in BMP- 2/4/7+ cell population was determined by immunofluorescence staining. The NG2+/BMP- 2+ cells were higher in the Agm treated group compared with EC group at (A) 7 and (B) 35 DPI. (C) Conversely the expansion of NG2+/BMP- 7+ cells were increased around the lesion site in Agm treated group at 35 DPI compared to EC group. (D) NG2+/BMP- 4+ cell population was decreased at 7 days and the (E) NG2+/BMP- 4+ cells were almost disappeared in the Agm treated group at 35 DPI. (TIF) [file pone.0053911.s008.tif]
