# Supplementary material for: The Multifaceted Effects of Agmatine on Functional Recovery after Spinal Cord Injury through Modulations of BMP-2/4/7 Expressions in Neurons and Glial Cells
Source: PLoS One. 2013 Jan 21;8(1):e53911. doi: 10.1371/journal.pone.0053911 (PMC3549976; doi:10.1371/journal.pone.0053911)
Supplement: Table S1 — The table showing the allocation of animals in normal control (NC), saline treated mice (EC group) and Agm treated mice (Agm treated group) following SCI. (DOCX) [file pone.0053911.s009.docx]

| **Groups** | **Technique** | **1 DPI** | **7 DPI** | **14 DPI** | **35 DPI** |
| --- | --- | --- | --- | --- | --- |
| **NC (*n*=78)** | **Western blot** | 4 | 4 | 4 | 4 |
|  | **Histology (CAST)** | 12 | 12 | 12 | 12 |
|  | **IHC/LFB** | 3 | 3 | 3 | 3 |
|  | **TEM** |  |  | 2 |  |
| **EC**  **(*n*=101)** | **Western blot** | 5 | 5 * | 5 * | 5 |
|  | **Histology (CAST)** | 15 | 15 | 15 ^#^ | 15 ^#^ |
|  | **IHC/LFB** | 4 | 4 | 5 | 5 |
|  | **TEM** |  |  | 3 |  |
| **Agm**  **(*n*=101)** | **Western blot** | 5 | 5 * | 5 * | 5 |
|  | **Histology (CAST)** | 15 | 15 | 15 ^#^ | 15 ^#^ |
|  | **IHC/LFB** | 4 | 4 | 5 | 5 |
|  | **TEM** |  |  | 3 |  |

**Table S1.**

*, The residual urine volume was performed in the same animals for western blot at 14 DPI.

^#^, The functional assessment was performed in the same animals used for histology at 35 DPI.
