# Supplementary material for: The Multifaceted Effects of Agmatine on Functional Recovery after Spinal Cord Injury through Modulations of BMP-2/4/7 Expressions in Neurons and Glial Cells
Source: PLoS One. 2013 Jan 21;8(1):e53911. doi: 10.1371/journal.pone.0053911 (PMC3549976; doi:10.1371/journal.pone.0053911)
Supplement: Table S2 — The average numbers of BMP- 2/4/7+ cells in neurons and glial cells in Th 8–Th 10 segments following SCI. The table showing the total number of BMP- 2/4/7+ cells co-localized with MAP-2+,Olig-2+ and GFAP+ cell population in Th 8–Th 10 segments at 1, 7, 14, and 35 DPI in NC group (n = 5), EC group (n = 5) and Agm treated group (n = 5) using computer assisted stereological toolbox (CAST) analysis. The immunopositive cells were measured per mm3 (Units:×106cells/mm3). †, p<0.05 NC group vs EC group; #, p<0.05 NC group vs Agm treated group; *, p<0.05 EC group vs Agm treated group. Results represent mean ± S.E.M. (DOCX) [file pone.0053911.s010.docx]

|  |  | **Neurons** | | **Oligodendrocytes** | |
| --- | --- | --- | --- | --- | --- |
|  |  | **EC** | **Agm** | **EC** | **Agm** |
| **BMP-2** | **1 DPI** | 14.01 ± 0.77 ^†^ | 19.00 ± 0.16 ^#^ | 1.01 ± 0.02 ^†^ | 1.30 ± 0.01 ^#^ |
|  | **7 DPI** | 18.07 ± 0.10 ^†^ | 25.08 ± 0.36 ^#,^ * | 1.74 ± 0.03 ^†^ | 2.41 ± 0.04 ^#,^ * |
|  | **14 DPI** | 10.49 ± 1.00 ^†^ | 15.46 ± 0.47 ^#^ | 0.04 ± 0.01 ^†^ | 1.25 ± 0.00 ^#,^ * |
|  | **35 DPI** | 5.02 ± 0.16 ^†^ | 10.01 ± 0.17 ^#,^ * | 0.05 ± 0.01 ^†^ | 0.70 ± 0.00 ^#^ |
|  | **NC** | 38.02 ± 0.16 | | 3.50 ± 0.01 | |
| **BMP-7** |  | **Neurons** | | **Oligodendrocytes** | |
|  |  | **EC** | **Agm** | **EC** | **Agm** |
|  | **1 DPI** | 0.59 ± 0.01 | 0.80 ± 0.00 ^#^ | 0.02 ± 0.00 ^†^ | 0.06 ± 0.00 |
|  | **7 DPI** | 1.10 ± 0.01 ^†^ | 1.51 ± 0.01 ^#,^ * | 0.02 ± 0.00 ^†^ | 0.08 ± 0.00 * |
|  | **14 DPI** | 0.80 ± 0.00 ^†^ | 1.20 ± 0.01 ^#,^ * | 0.03 ± 0.00 | 0.06 ± 0.00 |
|  | **35 DPI** | 0.41 ± 0.00 | 0.51 ± 0.00 | 0.03 ± 0.00 | 0.04 ± 0.00 |
|  | **NC** | 0.28 ± 0.00 | | 0.12 ± 0.01 | |
| **BMP-4** |  | **Astrocytes** | | **Oligodendrocytes** | |
|  |  | **EC** | **Agm** | **EC** | **Agm** |
|  | **1 DPI** | 0.75 ± 0.00 | 0.65 ± 0.01 | 0.23 ± 0.00 | 0.21 ± 0.00 |
|  | **7 DPI** | 1.70 ± 0.01 ^†^ | 1.00 ± 0.02 * | 0.42 ± 0.00 | 0.38 ± 0.01 |
|  | **14 DPI** | 2.45 ± 0.02 ^†^ | 1.60 ± 0.01 ^#,^ * | 0.30 ± 0.00 | 0.15 ± 0.00 * |
|  | **35 DPI** | 1.65 ± 0.02 ^†^ | 1.30 ± 0.02 | 0.28 ± 0.01 | 0.15 ± 0.00 * |
|  | **NC** | 0.90 ± 0.01 | | 0.30 ± 0.01 | |

**Table S2.**

Data are means ± S.E.M.

†, *P* ‹ 0.05 for NC group vs EC group; #, *P* ‹ 0.05 for NC group vs Agm group; *, *P* ‹ 0.05 for EC vs Agm group

NC: normal control group; EC: experimental control group; Agm: agmatine treated group; DPI: days post injury

Cell number: x 10^6^ cells/mm^3^; *n*=5 per group
